# The influence of endurance exercise training on myocardial fibrosis and arrhythmogenesis in a coxsackievirus B3 myocarditis mouse model

**DOI:** 10.1038/s41598-024-61874-x

**Published:** 2024-06-02

**Authors:** Kasper Favere, Manon Van Hecke, Sander Eens, Matthias Bosman, Peter L. Delputte, Johan De Sutter, Erik Fransen, Tania Roskams, Pieter-Jan Guns, Hein Heidbuchel

**Affiliations:** 1 Laboratory of Physiopharmacology, GENCOR, University of Antwerp, 2610 Antwerp, Belgium; 2 Research Group Cardiovascular Diseases, GENCOR, University of Antwerp, 2610 Antwerp, Belgium; 3 Department of Cardiology, Antwerp University Hospital, 2650 Antwerp, Belgium; 4 Department of Internal Medicine, Ghent University, 9000 Ghent, Belgium; 5 Translational Cell and Tissue Research, Department of Imaging and Pathology, University of Leuven, 3000 Leuven, Belgium; 6 Laboratory of Microbiology, Parasitology and Hygiene, University of Antwerp, 2610 Antwerp, Belgium; 7 Centre for Medical Genetics, University of Antwerp, 2610 Antwerp, Belgium

**Keywords:** Viral myocarditis, Exercise, Myocardial fibrosis, Scarring, Inflammation, Arrhythmogenesis, Cardiovascular diseases, Ventricular tachycardia

## Abstract

Nonischaemic myocardial fibrosis is associated with cardiac dysfunction, malignant arrhythmias and sudden cardiac death. In the absence of a specific aetiology, its finding as late gadolinium enhancement (LGE) on cardiac magnetic resonance imaging is often attributed to preceding viral myocarditis. Athletes presenting with ventricular arrhythmias often have nonischaemic LGE. Previous studies have demonstrated an adverse effect of exercise on the course of acute viral myocarditis. In this study, we have investigated, for the first time, the impact of endurance training on longer-term outcomes such as myocardial fibrosis and arrhythmogenicity in a murine coxsackievirus B3 (CVB)-induced myocarditis model. Male C57BL/6J mice (n = 72) were randomly assigned to 8 weeks of forced treadmill running (EEX) or no exercise (SED). Myocarditis was induced 2 weeks later by a single intraperitoneal injection with CVB, versus vehicle in the controls (PBS). In a separate study, mice (n = 30) were subjected to pretraining for 13 weeks (preEEX), without continuation of exercise during myocarditis. Overall, continuation of exercise resulted in a milder clinical course of viral disease, with less weight loss and better preserved running capacity. CVB-EEX and preEEX-CVB mice tended to have a lower mortality rate. At sacrifice (i.e. 6 weeks after inoculation), the majority of virus was cleared from the heart. Histological assessment demonstrated prominent myocardial inflammatory infiltration and cardiomyocyte loss in both CVB groups. Inflammatory lesions in the CVB-EEX group contained higher numbers of pro-inflammatory cells (iNOS-reactive macrophages and CD8^+^ T lymphocytes) compared to these in CVB-SED. Treadmill running during myocarditis increased interstitial fibrosis [82.4% (CVB-EEX) vs. 56.3% (CVB-SED); *P* = 0.049]. Additionally, perivascular and/or interstitial fibrosis with extensive distribution was more likely to occur with exercise [64.7% and 64.7% (CVB-EEX) vs. 50% and 31.3% (CVB-SED); *P* = 0.048]. There was a numerical, but not significant, increase in the number of scars per cross-section (1.9 vs. 1.2; *P* = 0.195), with similar scar distribution and histological appearance in CVB-EEX and CVB-SED. In vivo electrophysiology studies did not induce sustained monomorphic ventricular tachycardia, only nonsustained (usually polymorphic) runs. Their cumulative beat count and duration paralleled the increased fibrosis between CVB-EEX and CVB-SED, but the difference was not significant (*P* = 0.084 for each). Interestingly, in mice that were subjected to pretraining only without continuation of exercise during myocarditis, no differences between pretrained and sedentary mice were observed at sacrifice (i.e. 6 weeks after inoculation and training cessation) with regard to myocardial inflammation, fibrosis, and ventricular arrhythmogenicity. In conclusion, endurance exercise during viral myocarditis modulates the inflammatory process with more pro-inflammatory cells and enhances perivascular and interstitial fibrosis development. The impact on ventricular arrhythmogenesis requires further exploration.

## Introduction

In athletes presenting with ventricular arrhythmias, which are often asymptomatic and revealed during routine pre-participation evaluation, recent data have shown that a substantial proportion have underlying nonischaemic myocardial fibrosis (MF). As a genetic cause is excluded in most, the aetiology of these scars is presumed to be the result of prior (silent) myocarditis based on their localisation^1,2^. It is estimated that up to 5% of human viral infections are (often subclinically) complicated with myocardial involvement^3^. Although the majority of myocarditis cases follow a benign course with complete recovery, some patients develop MF as a consequence of cardiomyocyte loss and inflammation-associated profibrotic signalling^4,5^. Young age and male sex are known risk factors for MF development in the context of myocarditis^6^.

The explanation for the presence of nonischaemic MF in athletes, and especially the possible relation with prior myocarditis, remains to be further elucidated. Regular physical training leads to adaptive structural, functional and electrical cardiac remodelling, commonly referred to as the ‘athlete’s heart’^7^. Although these changes are considered to be physiological, there is evidence that (intensive) exercise can induce cardiac morbidity on itself, or can worsen pre-existing cardiac disease, for example right ventricular arrhythmogenic cardiomyopathy^8^. Increased wall stress has been implicated as a pathophysiologic driver^9^. In addition, it is well recognised that exercise affects the immune system and that the initial inflammatory phase during myocarditis may predispose to ventricular arrhythmias^10,11^. Therefore, sports guidelines recommend avoidance of intense sports during acute myocarditis (and/or pericarditis), and in some countries, athletes are even advised to abstain from sports during any acute viral infection^12^. A few studies were conducted in murine models to explore the impact of exercise on the early course of viral myocarditis, with the majority reporting an aggravating effect^13–19^. At present, no studies reported on longer-term impact, like MF or arrhythmias.

Our group recently raised the hypothesis that the presence of MF in athletes with arrhythmias may not merely be a coincidence, but that exercise itself might influence fibrogenesis during a (subclinical) viral infection, and may also lead to more profound proarrhythmogenic remodelling^1^. In the present study in mice, we mimic the athletic condition by forced treadmill running and combined it with the experimental coxsackievirus B3 (CVB) myocarditis model. Mice were subjected to pretraining, with and without continued training during a CVB infection. In contrast to previous studies, the effect of the exercise training was investigated on the long-term since we wanted to focus on MF and its association with ventricular arrhythmia.

## Material and methods

### Animals and ethical approval

A total of 72 wild-type C57BL/6J mice (male sex; age 11 weeks; mean weight 24.9 g (standard deviation 1.6 g); Charles River Laboratories, Belgium) were used for the first study, from now on referred to as the ‘continued EEX study’. Next, 30 wild-type C57BL/6J mice (male sex; age 11 weeks; mean weight 24.2 g (standard deviation 1.6 g); Charles River Laboratories, Belgium) were used for the second study, hereby designated as the ‘pretrained EEX study’. All mice were kept in standard cages (maximum 6 mice per cage) at the pathogenic unit of the University of Antwerp, at a constant environmental temperature of 22°C and humidity of 50% in a 12-h controlled light/dark cycle. The animals were fed a standard chow diet and had free access to food and drinking water. The groups were assigned at random. The study protocol has been approved by the University of Antwerp Ethical Committee for Animal Testing (file number 2019_33 for the continued EEX study and 2021_45 for the pretrained EEX study) and conforms with the guidelines from Directive 2010/63/EU of the European Parliament on the protection of animals used for scientific purposes, and with the Belgian Royal Decree of 2013 (C − 2013/24,221). This study is reported in accordance with the ARRIVE guidelines.

### Study design

The experimental workflow of the continued EEX study is schematically illustrated in Supplementary Fig. 1A. Mice were randomly assigned to 4 groups: coxsackievirus B3 (CVB) combined with exercise (CVB-EEX; exercise protocol see below) (n = 22), CVB without exercise (sedentary) (CVB-SED) (n = 22), control (i.e. phosphate-buffered saline-injected mice) combined with exercise (PBS-EEX) (n = 14) and PBS without exercise (PBS-SED) (n = 14). After 2 weeks of treadmill running (EEX groups) or not (SED groups), mice received intraperitoneal injection with CVB or PBS (D0). Afterwards, the exercise was continued for another 6 weeks in the EEX groups. Treadmill exhaustion testing was performed at three points in time: at the start of the study protocol (D-14), after two weeks of exercise training but prior to inoculation (D-1) and at the end of the study (D42). In a random selection of the animals (n = 10 for each of the CVB groups, n = 5 for each of the PBS groups), electrophysiology studies (EPS) were conducted immediately prior to sacrifice and organ dissection. Sacrifice took place from D43 to D50 post inoculation.

**Figure 1:**
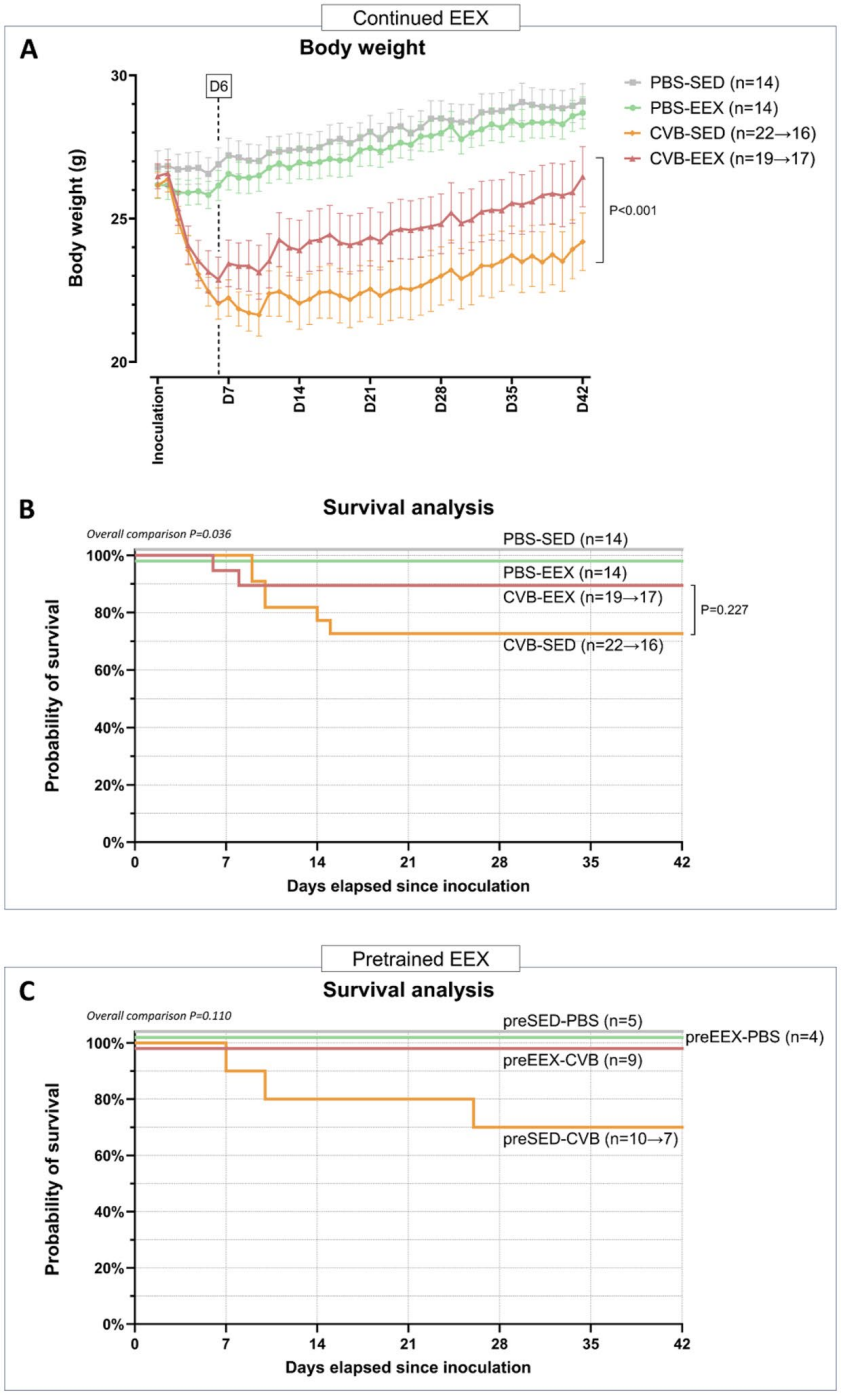
Clinical observations. (**A**) Serial body weight evolution. The body weight was assessed daily. The mean value for each group is shown. Error bars indicate the SEM. Piecewise linear regression models (accounting for a ‘knot’ where the slope alters), indicated that the change in body weight over time was different between the 4 groups. Refitting of the piecewise linear regressions demonstrated that the slopes of the PBS groups were not significantly different before and from day 6 on. However, the slopes of the CVB-EEX group did differ significantly from the slopes of the CVB-SED group, both before day 6 (-0.61 g/day vs. -0.86 g/day; *P* < 0.001) and after the knot at day 6 (+ 0.08 g/day vs. + 0.04 g/day; *P* < 0.001). (**B**) Kaplan–Meier survival analysis of the continued EEX study. To prevent visual overlap, the curves of PBS-SED and PBS-EEX have been slightly nudged (+ 2 and -2 data units on the Y-axis, respectively). A significant overall difference between the survival curves was observed (logrank (Mantel-Cox) test *P* = 0.036). Pairwise comparisons using Bonferroni correction detected no significant differences. (C) Kaplan–Meier survival analysis of the pretrained EEX study. To prevent visual overlap, the curves of preSED-PBS, preEEX-PBS, and preEEX-CVB have been slightly nudged (+ 4, + 2, and -2 data units on the Y-axis, respectively). The logrank (Mantel-Cox) test did not demonstrate differences between the survival curves.

The study design of the pretrained EEX study is shown schematically in Supplementary Fig. 1B. Mice were randomly assigned to 4 groups using a 2:2:1:1 allocation ratio: exercise training followed by inoculation with CVB (preEEX-CVB) (n = 10), no exercise followed by CVB inoculation (preSED-CVB) (n = 10), EEX with subsequent inoculation with PBS (preEEX-PBS) (n = 5), and SED followed by PBS injection (preSED-PBS) (n = 5). After 13 weeks of treadmill exercise training (preEEX groups) or not (preSED groups), animals received intraperitoneal injection with CVB or PBS (D0). From 24 h prior to inoculation (with virus or control), exercise training was discontinued in all groups till the end of the study. The mice were sacrificed at D44-D50 and were subjected to in vivo EPS immediately prior to sacrifice.

### Treadmill exercise training

**Figure 2:**
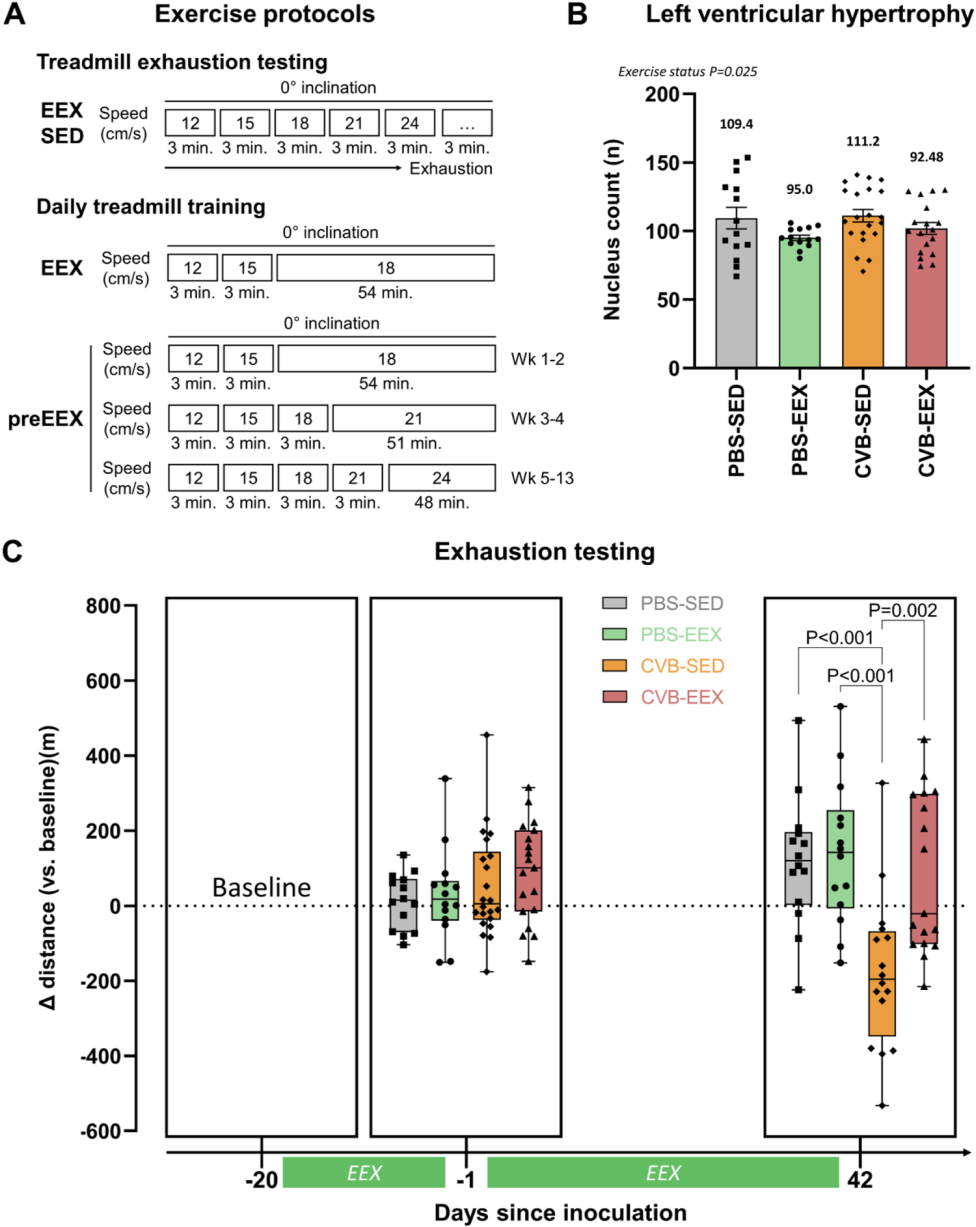
Exercise training. (**A**) Schematic overview of the exercise protocols. Serial treadmill exhaustion tests were performed in all groups of the continued EEX study. During these tests, the running speed was increased every 3 min until exhaustion occurred. The exercise groups (EEX and preEEX) were subjected to daily treadmill training. (**B**) Cardiac hypertrophy assessment via the ‘median cell count method’. Counts inversely correlate to cardiomyocyte size, with lower values thus indicating hypertrophy. The mean value for each group is displayed in absolute numbers. Statistical significance was tested by two-way ANOVA. There was no interaction between the factors (*P* = 0.632). Simple main effects analysis revealed a significant exercise effect (*P* = 0.025). Group size: PBS-SED: n = 14; PBS-EEX: n = 14; CVB-SED: n = 21; CVB-EEX: n = 18. (**C**) Treadmill exhaustion testing. The Y-axis indicates the difference in distance run compared to the first exhaustion test (baseline). Mixed-effects analysis, with an interaction time x group, indicated a difference in change over time between the groups (*P* < 0.0001). Analysis of the two follow-up time points separately, indicated no significant differences after the initial 2 weeks of training. At the study end, the exercise capacity of the CVB-SED group was decreased and significantly lower compared to the other groups (CVB-EEX vs. CVB-SED: *P* = 0.0019; PBS-EEX vs. CVB-SED: *P* = 0.0003; PBS-SED vs. CVB-SED: *P* = 0.0008; one-way ANOVA and post hoc analysis with Tukey correction). Group size: PBS-SED: n = 14; PBS-EEX: n = 14; CVB-SED: n = 22 (D-20 & D-1) → n = 16 (D42); CVB-EEX: n = 22 (D-20) → n = 19 (D-1) → n = 17 (D42).

Exercise training was performed on a motorised 5-lane treadmill with shock grid (LE8710MTS, Panlab, Barcelona, Spain). The shock grid administers a small electric shock to the hindlimb on contact to ensure that the animals run effectively. The shock intensity was set at 0.5 mA. The zone comprising the shock grid and one body length in front of the grid was defined as the ‘fatigue zone’. Mice failing to move out of the fatigue zone during training, and therefore residing there for ≥ 5 consecutive seconds, were removed from the training session and excluded from the study. Mice requiring > 4 shocks per minute of running to continue the training were also excluded from further study participation^20^. The front end of the treadmill was covered by a cloth, creating a dark environment and providing additional encouragement to avoid the back portion of the treadmill^21^. All mice had training sessions for 5 consecutive days per week, followed by two days without exercise intervention. Every training session started with a warm-up period during which the running speed was increased per 3 cm/s every 3 min from 12 cm/s to the target speed . In the continued EEX study, the target speed was set at 18 cm/s for the entire study duration. In the pretrained EEX study, the target speed was increased throughout the study from 18 cm/s (weeks 1–2 of the 13-week exercise programme) to 21 cm/s (weeks 3–4), and finally 24 cm/s. Each training session comprised 60 min of running including warm-up (Fig. 2A). Exercise training was consistently performed in the early morning in a silent environment without presence of other animal species. One investigator (KF) constantly observed the treadmill sessions to ensure effective running. The exercise protocols were designed based on an in-depth study of the literature and in-house expertise^20,22, 23^.

### Treadmill exhaustion test

In the continued EEX study, serial treadmill exhaustion tests were performed. Exhaustion testing was preceded by a minimum 48-h exercise abstinence period for the exercise groups. Sedentary mice (and all mice at the start of the study) were familiarised with the treadmill by placing them on the stationary belt and subsequently letting them walk at low speed (5 cm/s) for 3 min, and this for 3 consecutive days prior to the testing^20^. Exhaustion testing was performed for each mouse separately. The incremental exercise test started at a speed of 12 cm/s, which was increased by 3 cm/s every 3 min until exhaustion (Fig. 2A). Exhaustion was defined as residing for at least 5 s in the ‘fatigue zone’. Again, testing was performed in a reserved environment.

### Virus culture, quantification and inoculation

Human coxsackievirus B3 (Nancy strain) was cultured and subsequently functionally quantified using a plaque assay. We refer to the Supplementary material both for details on virus culture and virus quantification. Mice were inoculated with 5 × 10^5^ plaque forming units (PFU) coxsackievirus B3 through intraperitoneal injection in the right lower quadrant. The inoculum was diluted in sterile PBS to obtain an injection volume of 500 µL per animal. Control animals received intraperitoneal injection of 500 µL sterile PBS.

### Cardiac electrophysiology study

Programmed electrical stimulation was performed via a transjugular right ventricular approach as previously described by our research group^24^. In brief, an octapolar catheter was positioned in the right ventricle. Subsequently, burst and ramp stimulation protocols were performed to assess arrhythmogenicity. The stimulation protocols are provided as Supplementary material. For assessment of arrhythmia inducibility and the cumulative arrhythmia burden, only tachycardia episodes of ≥3 consecutive ventricular beats were taken into account (either nonsustained, *i.e.* spontaneous termination within 30 s, or sustained ventricular tachycardia (NSVT or VT)). Arrhythmia inducibility refers to whether, and at which step, the aforementioned ventricular arrhythmias could be elicited. The cumulative arrhythmia burden reflects the summed duration of these arrhythmias throughout the entire protocol.

### Euthanasia and tissue prelevation

Prior to euthanasia, a 48-h exercise abstinence period was introduced to avoid interference from acute effects induced by the last running session. Mice were anaesthetised via single intraperitoneal injection of pentobarbital sodium (150 mg/kg; Sanofi, Belgium) (if no prior EPS) or Avertin® (2,2,2-tribromoethanol; 250 mg/kg) (if EPS prior to sacrifice). Avertin® offers the advantage that a rapid and stable plane of surgical anaesthesia can be achieved by a single injection. It does not require additional equipment (such as compressed oxygen or dispensers). In addition, the impact on the cardiac physiology is limited compared to other anaesthetics^25,26^. Animals were euthanised by exsanguination under deep anaesthesia, as confirmed by the absence of tail and toe pinch reflexes. Upon prelevation, the heart was immersed in ice-cold PBS with 30 mmol/L KCl to arrest the cardiomyocytes in diastole, and remove blood and/or any debris. All biopsies for routine histology and immunohistochemistry were fixated in 4% neutral buffered (pH 7.2) formaldehyde solution for approximately 24 h, and subsequently transferred to PBS until embedding in paraffin. For additional molecular biology testing in the continued EEX study, the cardiac apex was immediately snap-frozen in liquid nitrogen and stored at − 80 °C until further use.

### Histology

For histologic assessment, 5 µm slices were prepared of each tissue paraffin block. The following stains were applied to all heart sections: hematoxylin and eosin (HE; 3801540BBE and 3801590BBE, Leica Biosystems, Wetzlar, Germany) and Picrosirius red (PSR, without counterstain; 09400-25, Polysciences, Hirschberg an der Bergstraße, Germany and 84512.260, VWR, Radnor, PA, USA). Two trained pathologists (TR and MVH) independently assessed the severity of myocardial inflammation (based on HE staining) and fibrosis (based on PSR staining) on a single cross-section of each mouse heart, using an in-house developed semiquantitative scoring system (Supplementary Figs. 2 and 3). For inflammation, the following categories were considered: absence of inflammation, perivascular inflammatory infiltration, interstitial inflammatory infiltration, interstitial inflammatory infiltration with focal cell loss, interstitial inflammatory infiltration with confluent cell loss. For fibrosis: perivascular (PV) fibrosis by which is meant the area surrounding the larger blood vessels (absent, limited (< 50% of the vessels) or extensive (> 50% of the vessels)), interstitial (IS) fibrosis by which is meant the space in between individual cardiomyocytes (absent, limited or extensive) and myocardial scarring by which is meant a continuous patch of fibrosis without intermittent cardiomyocytes (absent or present) were scored separately since these categories correlate to the aetiologic mechanism of the fibrosis pattern. Additionally, the location of each lesion was specified (left or right ventricle, subendocardial, midmyocardial of subepicardial). Scoring was performed in a blinded fashion. The interobserver agreement (expressed as weighted kappa coefficient) for the initial histopathological scoring of inflammation, PV fibrosis and IS fibrosis was 73.7%, 61.1% and 72.5%, respectively. For the myocardial scar count, the intraclass correlation coefficient was 77%. These coefficients are comparable to the coefficients reported in the context of liver disease assessment^27,28^. Subsequently, scoring discrepancies were discussed and the consensus scoring was established (still blinded from study group assignment) and used for final analysis.

Quantitative assessment of the extent of inflammation and fibrosis was performed by a blinded, trained pathologist (MVH). HE and PSR slides were scanned (Zeiss Axio Scan.Z1, Oberkochten, Germany) and imported into QuPath software (version 0.3.2) for image analysis^29^. Areas of dense inflammation were manually delineated, and subsequently added up to estimate the total inflammation area. For fibrosis quantification, the ventricular lumina and any possible regions of false-positive staining were manually excluded. Next, a well-considered software threshold was set for the entire batch to calculate the total PSR-positive area. The total inflammatory and PSR-positive area were divided by the respective total heart area to obtain the percentage of inflammation- and PSR-positivity for each animal, respectively.

Cardiac hypertrophy assessment was performed on digitalised HE slides (Zeiss Axio Scan.Z1, Oberkochten, Germany) using QuPath software (version 0.3.2)^29^. In four regions of the left ventricle (anterior, lateral, inferior and septal), a zone with cross-sectional orientation of the cardiomyocytes and without evident inflammation, cell loss or fibrosis was identified by a blinded, trained pathologist (MVH). The number of cell nuclei (indicative of cardiomyocytes) in a square of exactly 40,000 µm^2^ in each of these 4 zones was automatically counted by the software. Subsequently, for each of the animals, the median of the 4 nuclei counts was taken. In this ‘median cell count method’, counts inversely correlate to the cardiomyocyte volume, and lower values therefore indicate hypertrophy.

For immunohistochemical characterisation of the inflammatory infiltrate, a selection of representative sections of CVB-EEX and CVB-SED mice were stained with the following commercially available antibodies using a peroxidase technique: anti-CD3 (ab16669, Abcam, Cambridge, UK), anti-CD4 (14-9766-82, ThermoFisher, Waltham MA, USA), anti-CD8 (14-0808-82, ThermoFisher, Waltham MA, USA), anti-F4/80 (ab6640, Abcam, Cambridge, UK), anti-iNOS (ab15323, Abcam, Cambridge, UK) and anti-Arginase1 (sc20150, Santa Cruz Biotechnology, Dallas TX, USA). Manual counting of these cell surface markers was performed by a blinded, trained pathologist (TR) on inflammation areas covering an entire microscopic high-power field (HPF × 400) (number of immunoreactive cells/HPF).

### RNA extraction

In the continued EEX study, total RNA was isolated from the cardiac apex using the RNeasy fibrous tissue mini kit (Qiagen GmbH, Hilden, Germany). The manufacturer’s instructions were followed. The tissue was mechanically homogenised with pellet pestles (Fisherbrand, Waltham MA, USA) driven by a mixer motor. The RNA purity and concentration were determined using a NanoDrop ND-2000 spectrophotometer (ThermoFisher, Waltham MA, USA). RNA was stored at − 80 °C as single use aliquots for further analyses.

### Quantitative PCR (qPCR)

For relative quantification of RNA expression in the continued EEX study, one-step qPCR was performed using the TaqMan fast virus 1-step master mix (ThermoFisher, Waltham MA, USA) combined with Taqman probes (ThermoFisher, Waltham MA, USA) according to the manufacturer’s instructions. Plates were run on a QuantStudio 3 cycler (ThermoFisher, Waltham MA, USA). For normalisation, the following reference gene combination was used: GAPDH (glyceraldehyde-3-phosphate dehydrogenase) (Mm99999915_g1), Cdkn1b (cyclin-dependent kinase inhibitor 1b) (Mm00438168_m1) and HPRT (hypoxanthine phosphoribosyltransferase) (Mm03024075_m1). Reference gene stability was assessed via the geNorm module of qbase + software (Biogazelle, Ghent, Belgium). The following target genes were determined: CTGF (connective tissue growth factor) (Mm01192933_g1), TGF-β (transforming growth factor β) (Mm01178820_m1), COL1A1 (Mm00801666_g1), COL3A1 (Mm00802305_g1) and MMP9 (matrix metalloproteinase 9) (Mm00442991_m1). Gene expression data analysis (including quality control) was performed in qbase + software (Biogazelle, Ghent, Belgium).

### Digital PCR (dPCR)

As part of the continued EEX study, samples were selected at random from the 4 groups for evaluation of the cardiac viral load via the QIAcuity One Digital PCR system (Qiagen GmbH, Hilden, Germany). The system was used in conjunction with QIAcuity nanoplate 26k 24-well plates (Qiagen GmbH, Hilden, Germany) and the QIAcuity one-step viral RT-PCR kit (Qiagen GmbH, Hilden, Germany). The CVB genome was detected with the following primer–probe combination: forward primer 5’-GGTGCGAAGAGTCTATTGAGC-3’, reverse primer 5’-CACCCAAAGTAGTCGGTTCC-3’ and probe 5’-/56-FAM/AATGCGGCT/ZEN/AATCCTAACTGCGGA/3IABkFQ/-3’ (IDT, Leuven, Belgium). Analysis was performed in QIAcuity Software Suite (version 2.0.20).

### Statistical analysis

In bar charts, bars represent the mean value and errors bars depict the standard error of the mean (SEM). In the box-and-whiskers plots, the horizontal line indicates the median, the top and bottom of the box the interquartile range, and the whiskers the range. Statistical analyses were performed using GraphPad Prism (version 9.4.0), SPSS Statistics (version 28) and R (version 4.2.1) software. Details on the statistical tests used are provided in the corresponding text paragraph or Figure legend. If required, normality was assessed by means of the D’Agostino & Pearson test. Statistical expertise was provided by Erik Fransen, Master in Statistical data analysis.

## Results

### Continued exercise attenuates weight loss in virally inoculated mice

Starting from three days after inoculation, virally infected animals began to show negative signs of health (reduced spontaneous mobility, hunched posture, ruffled coat) and weight loss (Fig. 1A). The negative signs of health became more prominent between days four and seven after inoculation, without difference between the exercising (preEEX or EEX) and non-exercising group. The adverse health signs gradually diminished from approximately two weeks after inoculation, but nevertheless remained present to some extent in the majority of virally infected animals till the end of the study.

The body weight evolution followed a similar trend as the clinical observations with gradual weight recuperation in the virus groups. In the continued EEX study, the weight loss was less pronounced (before day 6: -0.61 g/day vs. -0.86 g/day; *P* < 0.001) and recovered faster (after day 6: +0.08 g/day vs. +0.04 g/day; *P* < 0.001) in the CVB-EEX group compared to the CVB-SED group. The pretrained EEX study did not demonstrate any mitigating effect of exercise pretraining on body weight (data not shown).

In the control groups, no mortality was observed. In the continued EEX study, mortality in the two CVB virally-infected groups occurred between D6 to D15 and was 11% and 27% for CVB-EEX and CVB-SED, respectively (*P* = 0.227) (Fig. 1B and Supplementary Table 1A). In the pretrained EEX study, between D7 to D26 post inoculation, 0% and 30% mortality was observed in the preEEX-CVB and preSED-CVB group, respectively (Fig. 1C and Supplementary Table 1B). The animals that died prematurely all showed severe myocarditis (*i.e.* with substantial confluent necrosis) on histology. Although the exact cause of death remains uncertain, we observed severe pancreatitis in CVB-infected animals of previous experiments.

### Continued exercise training prevents loss of exercise capacity in virally inoculated mice

In the continued EEX study, prior to inoculation, the exercise groups experienced no difficulties to comply with the exercise training. After inoculation, 3 animals (all virus-inoculated) were not capable to adequately complete the exercise protocol (excessive shocking and/or refusal to run) and were therefore excluded from the study. In the pretrained EEX study, by the end of the training programme (week 13), eventually 2 animals had to be excluded because of excessive shocks.

In the continued EEX cohort, exercise training induced mild but significant cardiomyocyte hypertrophy, as can be seen from Fig. 2B. Treadmill exhaustion testing showed no difference in exercise capacity between the groups after the first two weeks of training. However, after completion of the entire exercise programme, the exercise capacity in the CVB-SED group was dramatically lower (median -196 m) in comparison to the other groups (PBS-SED: +120 m; PBS-EEX: +142 m; CVB-EEX: -21 m) (Fig. 2C).

### Continued exercise alters the inflammatory response in viral myocarditis

**Figure 3:**
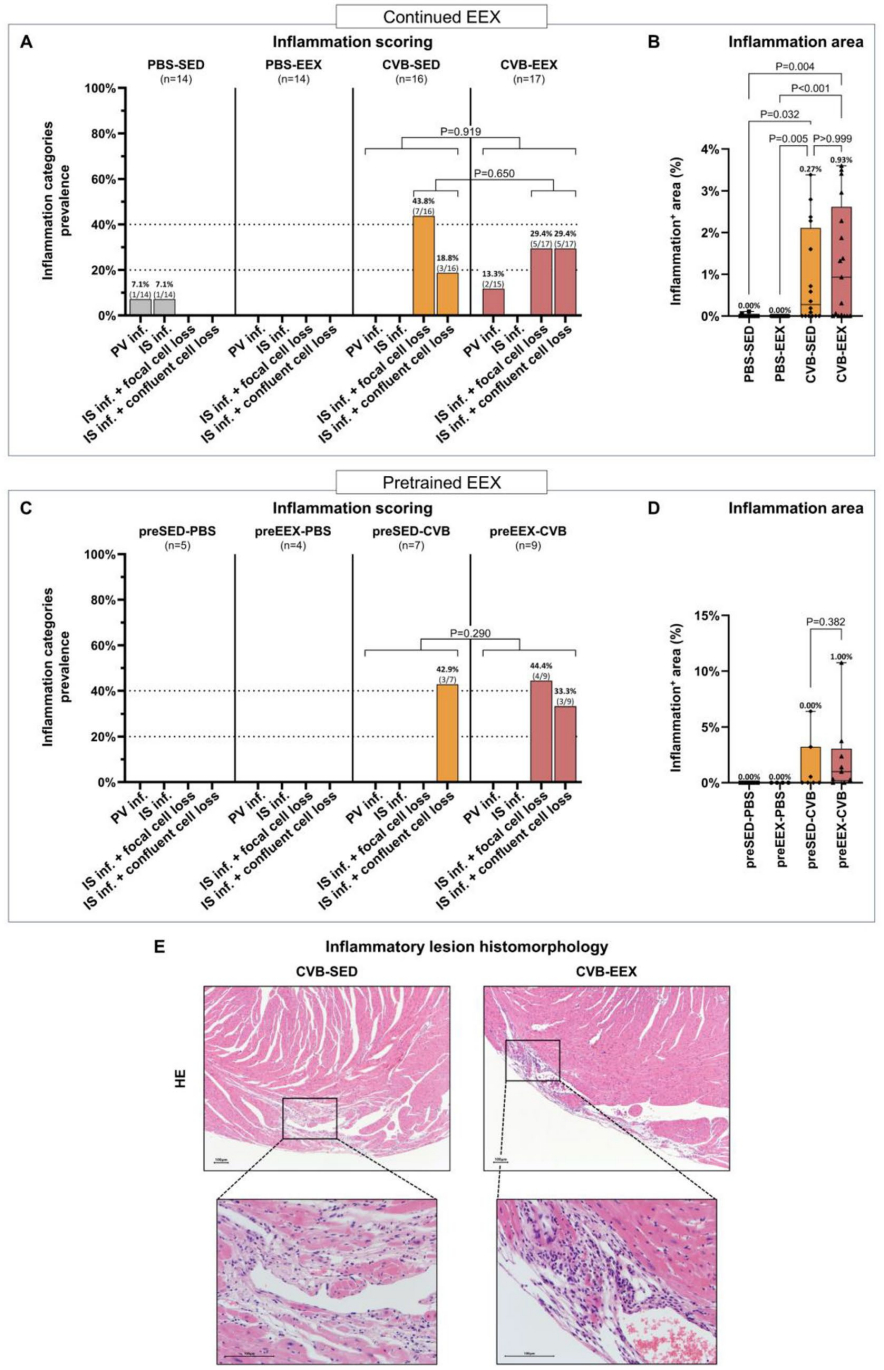
Myocardial inflammation at sacrifice. (**A**) Frequency histograms of semiquantitative inflammation scoring in the continued EEX study. The scoring distribution was significantly different between all groups in general (*P* < 0.001; Monte Carlo Pearson Chi-Square), but not between CVB-EEX and CVB-SED, neither for all inflammatory categories (*P* = 0.919; Monte Carlo linear-by-linear association), nor with regard to cardiomyocyte loss (focal vs. confluent) (*P* = 0.650; Monte Carlo Pearson Chi-Square). The odds ratio for confluent loss of cardiomyocytes was 2.33 (95% CI 0.37–14.71) in CVB-EEX compared to CVB-SED. Abbreviations: inf., infiltration; IS, interstitial; NS, not significant; PV, perivascular. (**B**) Inflammation area in the continued EEX study. The median values are displayed in numbers above each box-and-whiskers plot. The Kruskal–Wallis test was used for statistical analysis, followed by Dunn’s multiple comparisons test. Group size: PBS-SED: n = 14; PBS-EEX: n = 14; CVB-SED: n = 16; CVB-EEX: n = 17. (**C**) Semiquantitative inflammation scoring in the pretrained EEX study as represented by frequency histograms. The scoring distribution was compared between preEEX-CVB and preSED-CVB by means of the Monte Carlo linear-by-linear association test (*P* = 0.290). (**D**) Inflammation area in the pretrained EEX study. The median values are displayed in numbers above each box-and-whiskers plot. The Mann–Whitney U test was used for statistical analysis. Group size: preSED-PBS: n = 5; preEEX-PBS: n = 4; preSED-CVB: n = 7; preEEX-CVB: n = 9. (**E**) Morphological examples of inflammatory lesions in exercising and sedentary CVB mice in the continued EEX study. On HE staining, there was more cellularity of the lesions in the exercising (right panel) compared to the sedentary group (left panel). Evidence for organisation of the lesions (haemosiderin-laden macrophages and spindled interstitial cells) was seen in both groups.

At the time of sacrifice of the continued EEX study (6 weeks after CVB or sham inoculation), myocardial inflammatory infiltration in the sham groups was negligible, without any evidence of cardiomyocyte loss (Fig. 3A). The presence of a small number of inflammatory cells in the heart (both in humans and laboratory animals) is considered to be physiological^30,31^. In contrast, the majority of CVB-infected animals still exhibited myocardial inflammatory infiltration, and showed cardiomyocyte loss. The surface of the dense infiltrates covered a median of 0.27% to 0.93% of the myocardium (Fig. 3B), versus about 8% at the acute stage (day 7; Supplementary Fig. 4). Scoring did not indicate differences in the severity of the myocardial inflammation in the exercising and sedentary CVB groups. Also the proportion of the cardiac cross-section demonstrating inflammatory infiltration did not differ between the CVB groups.

**Figure 4:**
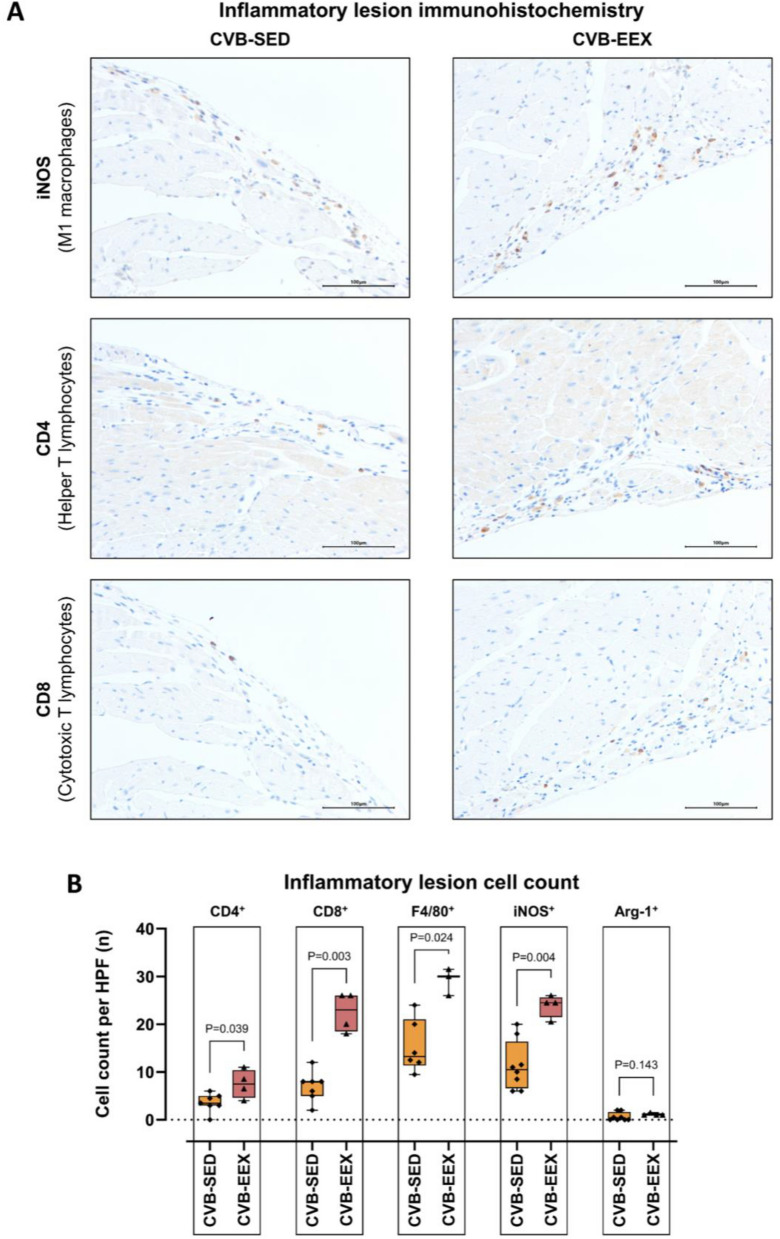
Immunohistochemical characterisation of inflammatory lesions in the continued EEX study. (**A**) Morphological examples of iNOS, CD4 and CD8 immunohistochemical staining of inflammatory lesions in CVB-EEX and CVB-SED animals. (**B**) Absolute cell count per high-power field (HPF) in inflammatory lesions of CVB-EEX and CVB-SED animals. If multiple lesions were considered for an animal, the counts were averaged, resulting in 1 datapoint for each animal studied. Group-by-group comparison was performed by means of the Mann–Whitney U test. Group size: CVB-SED: n = 8; CVB-EEX: n = 4.

The inflammatory lesions in the CVB groups were considered to be in a resolving, organising state, as evidenced by relatively low presence of immune cells and the detection of haemosiderin-laden macrophages and spindled fibroblasts (Supplementary Fig. 4 shows an early inflammatory lesion for reference). However, morphological and immunohistochemical characterisation revealed higher cellular density of the inflammatory infiltrates in the CVB-EEX group compared to the CVB-SED group (Fig. 3E and 4A, B). In both groups, the infiltrates consisted mainly of macrophages (F4/80 positive) and lymphocytes (CD3-positive T lymphocytes), with sparse plasma cells. Nevertheless, in the CVB-EEX group, the absolute numbers of CD8^+^ T lymphocytes and macrophages (in general (F4/80^+^), and the iNOS-reactive subtype in particular) per unit of inflammation area were higher.At the time of sacrifice of the pretrained EEX study, myocardial inflammation was no longer present in all virally infected animals. Scoring did not indicate differences in the severity of the myocardial inflammation between the pretrained and sedentary CVB groups (Fig. 3C). The proportion of the cardiac cross-section demonstrating inflammatory infiltration did not differ between the CVB groups either (Fig. 3D). Finally, the composition of the inflammatory infiltrate present in the cardiac lesions was similar between pretrained and sedentary CVB groups (data not shown).

### Virus is effectively cleared from the heart at day 43–50, irrespective of exercise training

**Figure 5:**
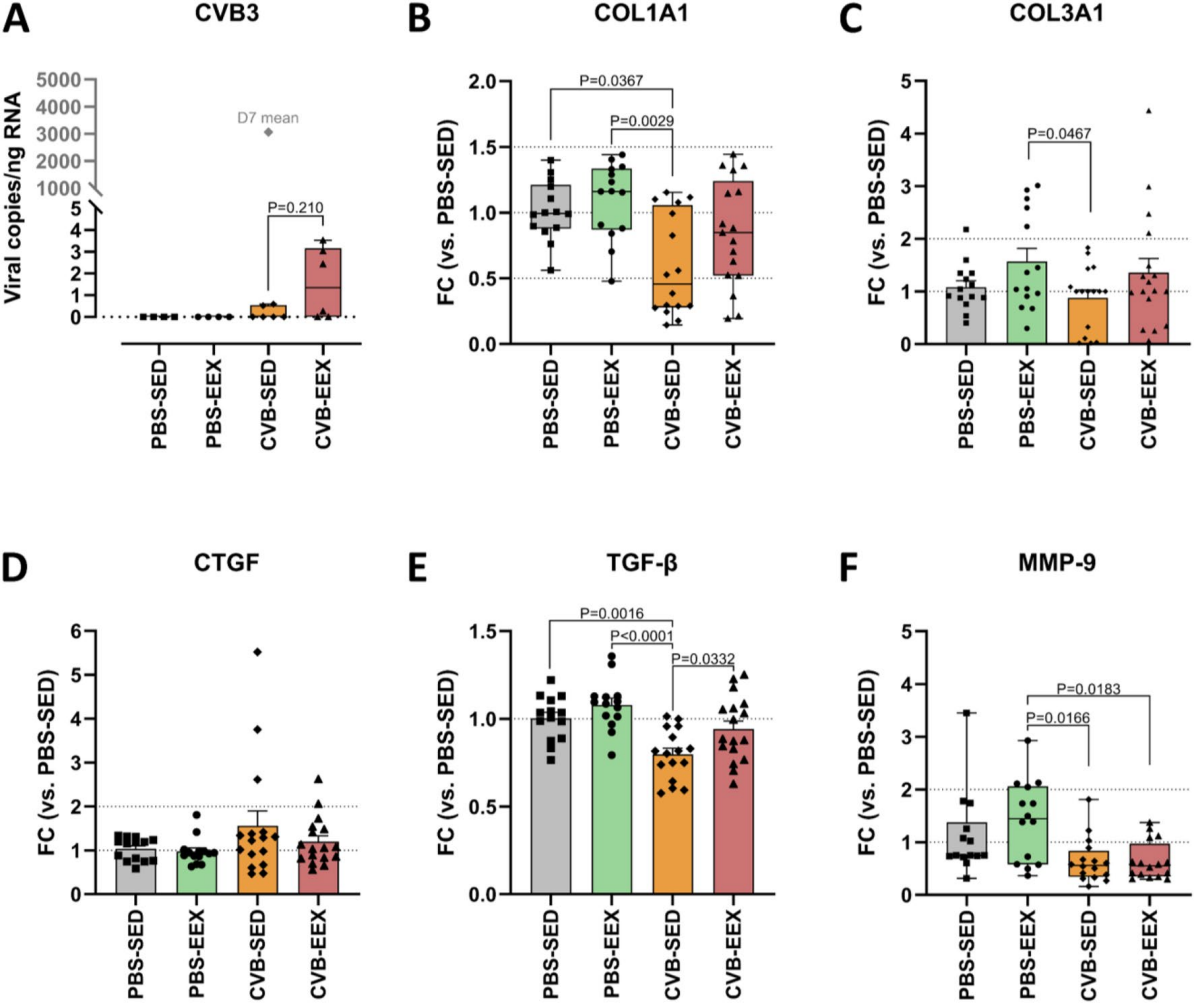
Cardiac PCR in the continued EEX study. (**A**) Cardiac CVB viral load at sacrifice as determined by dPCR. The viral load per group is expressed as copies per ng of RNA. A Mann–Whitney U test did not detect a significant difference between the CVB-EEX and CVB-SED group (*P* = 0.210). Group size: n = 4 for PBS groups and n = 6 for CVB groups. The mean myocardial viral load for similar animals (strain, age, sex, inoculation dose) at 7 days after inoculation is shown for reference. (**B**-**F**) Cardiac gene expression at sacrifice as determined by qPCR. Messenger RNA levels are expressed as the fold change (FC) over the PBS-SED control group. Potential differences in CTGF, TGF-β and COL3A1 gene expression were evaluated via one-way ANOVA with post hoc Tukey’s test. For comparison of MMP-9 and COL1A1 gene expression between groups, the Kruskal–Wallis test was used, followed by Dunn’s multiple comparisons test. Group size: PBS-SED: n = 14; PBS-EEX: n = 14; CVB-SED: n = 16; CVB-EEX: n = 16.

In accordance with the resolving inflammatory state on histology, PCR detected only very low viral loads in the CVB groups of the continued EEX study at the time of sacrifice (i.e. 43–50 days after inoculation) (Fig. 5A). For reference, in this model, the myocardial viral load 7 days after inoculation is on average > 3000 copies/ng RNA (with a viral myocarditis induction rate on histology of 100%) (unpublished in-house data). In addition, 2/6 CVB-EEX samples and 4/6 CVB-SED samples were virus-negative. The results indicate that the viral particles and virally infected cells have been almost completely eliminated 42 days after inoculation.

### Continued exercise increases cardiac interstitial fibrosis development in viral myocarditis

**Figure 6:**
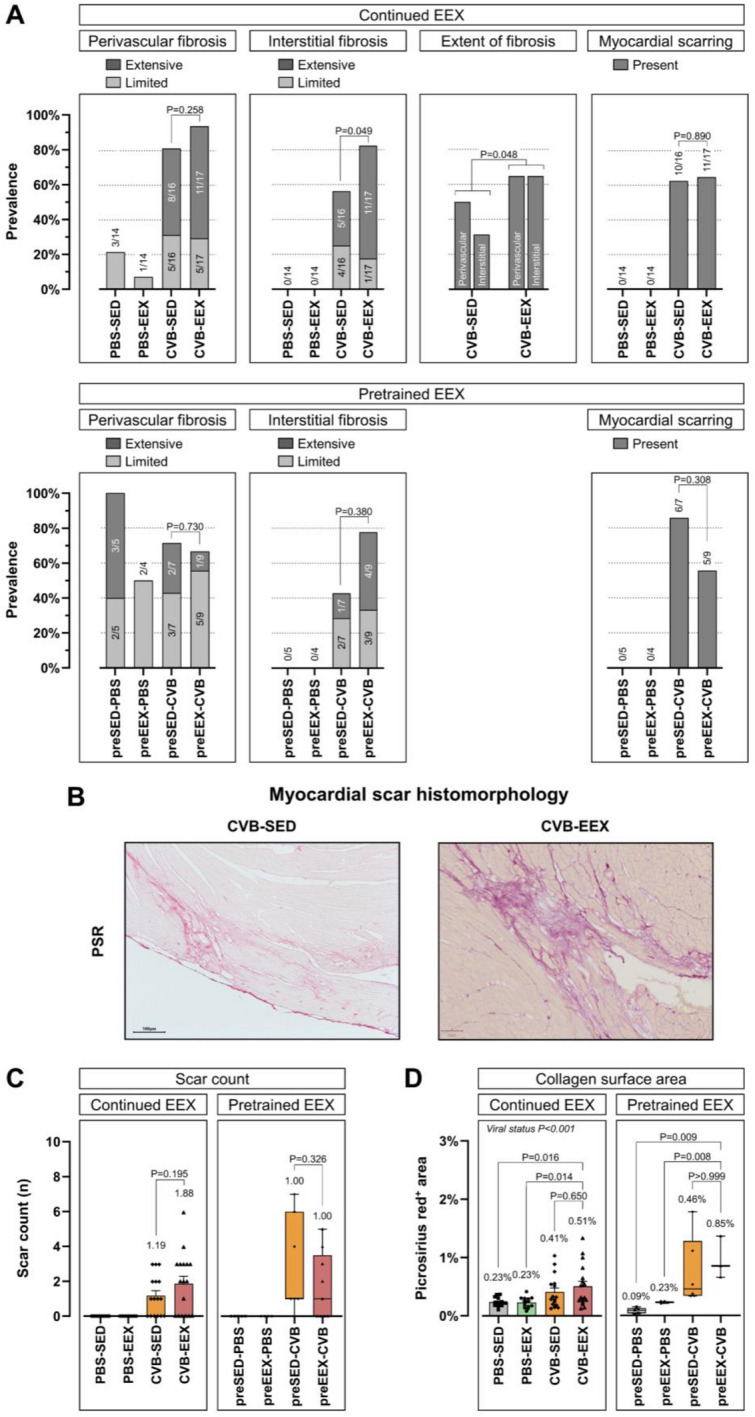
Myocardial fibrosis at sacrifice in continued EEX and pretrained EEX. (**A**) Semiquantitative fibrosis scoring at sacrifice. The fibrosis scoring is presented as frequency histograms. The corresponding prevalence ratios are also shown numerically. The different fibrosis categories were compared between the CVB animals using the Monte Carlo linear-by-linear association test (perivascular fibrosis, interstitial fibrosis), Chi-Square test (myocardial scarring in the continued EEX study), and Fisher’s exact test (myocardial scarring in the pretrained EEX study). For the continued EEX study, a logistic regression model showed that extensively distributed fibrosis within the perivascular and interstitial categories was significantly more likely to occur in the CVB-EEX group compared to the CVB-SED group (*P* = 0.048). (**B**) Morphological examples of fibrotic lesions of exercising and sedentary CVB mice in the continued EEX study. On PSR staining, the fibrotic zones appeared mature, with presence of densely organised collagen bundles, in both groups without noticeable differences. (**C**) Comparison of the myocardial scar count per cross-section between the CVB groups was performed by means of a Student’s *t*-test (continued EEX study) or Mann–Whitney U test (pretrained EEX study). Group size: PBS-SED: n = 14; PBS-EEX: n = 14; CVB-SED: n = 16; CVB-EEX: n = 17; preSED-PBS: n = 5; preEEX-PBS: n = 4; preSED-CVB: n = 7; preEEX-CVB: n = 9. (**D**) For the Picrosirius red-positive myocardial area, group comparison was conducted via two-way ANOVA followed by post hoc comparison with Tukey correction (continued EEX study, detecting a significant main effect for viral status (CVB vs. PBS) (*P* < 0.001)) or Kruskal–Wallis test, followed by Dunn’s multiple comparisons test (pretrained EEX study). Large areas of false-positive staining required the exclusion of several animals. Group size: PBS-SED: n = 14; PBS-EEX: n = 14; CVB-SED: n = 16; CVB-EEX: n = 17; preSED-PBS: n = 5; preEEX-PBS: n = 4; preSED-CVB: n = 6; preEEX-CVB: n = 3.

In the continued EEX study, the control groups generally did not exhibit myocardial fibrosis, except for a small fraction with (physiological) strands in the perivascular territory. In contrast, the majority of CVB-inoculated animals showed perivascular and interstitial fibrosis, which was often extensive. In addition, myocardial scarring following manifest cell death and repair was seen in a large proportion of the CVB mice. CVB-EEX animals had more interstitial fibrosis (*P* = 0.049) and fibrosis with extensive distribution in general (perivascular and/or interstitial category) (*P* = 0.048) compared to the CVB-SED animals (Fig. 6A). In the pretrained EEX study, scoring confirmed the presence of perivascular and interstitial fibrosis, but without significant differences between pretrained and sedentary mice (Fig. 6A).

The myocardial scar count was on average 1.2 in the CVB-SED group and 1.9 in the CVB-EEX group (*P* = 0.195) in the continued EEX study (Fig. 6C). In both groups, the majority of scars were located in the midmyocardium of the left ventricle, without particular predilection for any of the cardiac walls (Supplementary Fig. 5). Morphological comparison of fibrotic lesions in the CVB-EEX and CVB-SED groups revealed no noticeable differences (Fig. 6B). In the pretrained EEX study, the majority of both CVB groups exhibited myocardial scarring as well, often with multiple scars per cross-section (preEEX-CVB vs. preSED-CVB scar prevalence *P* = 0.308 and scar count *P* = 0.326).

Automated quantification of the Picrosirius red positive myocardial area in the continued EEX study showed slightly higher collagen content in the CVB animals, but no difference was detected between the CVB-EEX and CVB-SED group (Fig. 6D). This observation was also made in the pretrained EEX study.

### Collagen and fibrogenesis genes are not upregulated in the late phase of viral myocarditis

At the time of sacrifice (D43-D50), cardiac CTGF mRNA levels were comparable in all groups. TGF-ꞵ expression was lower in CVB-SED compared to the other groups. The COL1A1 and COL3A1 gene expressions were on average lowest in the CVB-SED group, and significantly lower compared to the PBS-EEX group, in which the expression was highest for both genes. MMP-9 gene expression followed the overall trend with expression being lower in the CVB-SED group, and also in the CVB-EEX group, compared to the PBS-EEX group (Fig. 5B-F).

### The combination of viral myocarditis and continued exercise is associated with the highest arrhythmia burden

**Figure 7:**
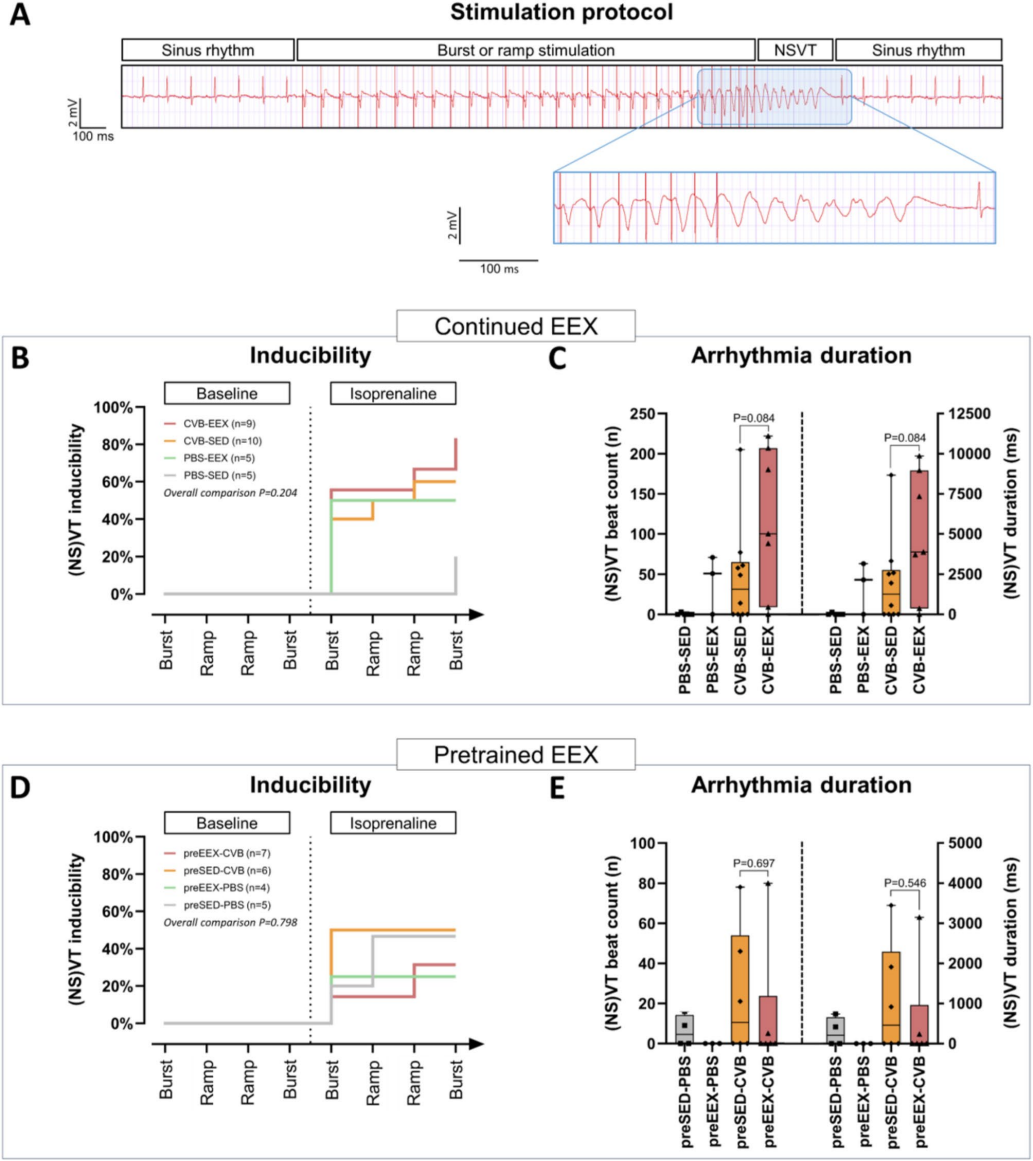
Ventricular arrhythmias (**A**) Illustration of the programmed electrical stimulation protocol. In the ECG tracing shown, ramp pacing elicits a NSVT episode of 7 beats. (**B**) Arrhythmia inducibility in the continued EEX study. Four stimulation steps were performed prior and after isoprenaline administration. No significant differences in inducibility were observed between the groups (logrank (Mantel-Cox) test *P* = 0.204). (**C**) Arrhythmia burden in the continued EEX study. The cumulative length of the induced arrhythmia episodes is expressed as the number of beats or duration in milliseconds. There was no significant difference between the CVB groups as evaluated by means of the Mann–Whitney U test. Three animals (2 from the CVB-EEX group and 1 from PBS-EEX) were excluded from the analysis because of periprocedural death, impeding completion of the stimulation protocol. Group size: PBS-SED: n = 5; PBS-EEX: n = 3; CVB-SED: n = 10; CVB-EEX: n = 7. (**C**) Arrhythmia inducibility in the pretrained EEX study. No significant differences with regard to inducibility were observed between the groups (logrank (Mantel-Cox) test *P* = 0.798). (**D**) Cumulative arrhythmia burden in the pretrained EEX study. The Mann–Whitney U test did not demonstrate significant CVB group differences. Animals not completing the entire stimulation protocol because of periprocedural death, were excluded from analysis (preSED-PBS: n = 1; preEEX-PBS: n = 1; preEEX-CVB: n = 1). Group size: preSED-PBS: n = 4; preEEX-PBS: n = 3; preSED-CVB: n = 6; preEEX-CVB: n = 6.

Increasingly aggressive stimulation protocols were delivered to a selection of the animals of each group before sacrifice, first at baseline and then after intraperitoneal injection of isoprenaline (Fig. 7A, B, D). The ventricular capture threshold was less than 100 µA in all animals. The mean ventricular effective refractory period (VERP) was 35 ms (range 21–47 ms) for the continued EEX study and 40 ms (range 30–62 ms) for the pretrained EEX study, without differences between groups (Kruskal–Wallis test* P* = 0.090 and* P* = 0.707, respectively).

At baseline, no (NS)VT episodes could be induced. In the continued EEX study, after administration of isoprenaline, ventricular arrhythmias were inducible in the majority of animals of the CVB-EEX (7/9) and CVB-SED (6/10) groups, and in 2/5 PBS-EEX animals and 1/5 PBS-SED animals, often already during the first stimulation step (Fig. 7B and Supplementary Table 2A). Supplementary Fig. 6 provides an overview of the duration of each of the ventricular arrhythmias induced for animals that completed the entire stimulation protocol. In the pretrained EEX study, after administration of isoprenaline, ventricular arrhythmias could be elicited in 2/7 animals of the preEEX-CVB group, 3/6 animals of preSED-CVB, 1/4 preEEX-PBS animals, and 2/5 preSED-PBS animals (Fig. 7D and Supplementary Table 2B). Sustained monomorphic ventricular tachycardia was never induced, only nonsustained (usually polymorphic) runs.

In the continued EEX study, the median cumulative duration of the arrhythmia episodes over the entire protocol, expressed as either total number of beats or as total duration, was over twofold higher in the CVB-EEX group (100 beats, corresponding to 3870 ms) than in the other groups and paralleled the fibrosis findings (CVB-SED: 31.5 beats/1255 ms; PBS-EEX: 51 beats/2157 ms) (Fig. 7C). The difference did not reach significance when CVB-EEX was compared with the CVB-SED group (*P* = 0.084 for beat count). In the pretrained EEX study, the cumulative burden of the arrhythmia episodes throughout the entire stimulation protocol was generally low (median arrhythmia burden per group 0–10.5 beats) (Fig. 7E). However, in the CVB groups, some animals experienced a relatively high ventricular arrhythmia burden (over fourfold higher than the highest burden recorded in the PBS groups). No significant difference was observed between the CVB groups (*P* = 0.697 for cumulative beat count). For both studies, the average heart rate of the arrhythmias was similar across the groups. Consequently, the trends remained unchanged when the arrhythmia burden was expressed as cumulative duration (instead of cumulative beat count).

## Discussion

In this study, we demonstrate that continued endurance exercise training had several effects in a murine model of coxsackieviral myocarditis. Firstly, the clinical disease course in the exercising mice was characterised by less weight loss and better preserved exercise capacity. Secondly, the inflammatory response was altered, with increased presence of pro-inflammatory cells in the lesions of exercising mice. Importantly, exercising mice demonstrated more extensive perivascular and interstitial fibrosis compared to their sedentary counterparts. Lastly, the increased fibrosis in the exercising CVB group may explain the tendency for a higher ventricular arrhythmia burden. These differences were no longer observed in mice who were submitted to pretraining prior to infection only, and stopped exercising on infection.

The impact of training in our continued EEX study was confirmed by the modest but significant myocardial hypertrophy, and mainly by the preservation of running distance despite myocarditis. The exhaustion test did not show increased exercise capacity in control mice. The moderate intensity of the training stimulus may explain this: we used a running speed of 18 cm/s for training, corresponding to approximately 75% of $\dot {\text {V}}\text O_2$max and 40% of maximum running speed^22,32^. We would therefore classify our training regimen as moderate-intensity continuous training^33,34^. It is known that the C57BL/6J strain is relatively resistant to exercise-induced physiological adaptations^35^. In future studies, we consider to measure oxygen consumption ($\dot {\text {V}}\text O_2$), a technique which may offer more resolution to detect training effects in this strain of mice.

The modulating effects of exercise on the course of viral myocarditis likely depend on a number of exercise characteristics (modality, intensity, duration and temporal relationship). Supplementary Table 3 summarises previous interventional studies on this subject. Concerning the training modality, we opted for treadmill running because it is a reliable, well-controlled and often superior model of exercise training for research studies^35^. A recent meta-analysis of animal studies showed that exercise intensity affects morbidity and mortality in non-cardiac viral infections^36^. We consider our moderate-intensity training protocol as more ‘realistic’ compared to previously used exercise intensities, since also athletes perform most of their training regimen at such intensity^37^. Concerning duration, we wanted our model to reflect endurance athletes. Therefore, we considered a period of pre-training for exercise habituation and physiological adaptations (although also from prior mice studies it remains unclear how much pre-training is required) and continued training during and after the acute phase of myocarditis. The fact that we did not observe differences in the pretrained EEX study, suggests that the modulating effects of exercise in this context subside rather quickly after discontinuation of the training. We conducted our study in the C57BL/6J strain because it is considered to be ‘resistant’ to viral myocarditis in the sense that the virus is eliminated, the cardiac damage is controlled and self-limiting, and they do not develop chronic myocarditis^38^. This most likely corresponds to the majority of human patients, especially given the large number of subclinical cases with complete recovery that never come to medical attention^4^.

Histologically, myocarditis led to both myocardial scarring, which is caused by manifest cell death and repair at a cellular level, as to more reactive patterns of fibrosis such as perivascular and interstitial fibrosis. Of the infected animals, the exercising group exhibited slightly, but significantly, more interstitial fibrosis and reactive fibrosis with extensive distribution in general. Furthermore, the 58% increase (albeit not statistically significant) in the average number of scars in the exercising group compared to the sedentary group, certainly requires further study. These histologic findings suggest for the first time that performing moderate-intense exercise during viral myocarditis promotes myocardial fibrosis development, and is confirmed by the absence of these changes in the pretrained EEX study. Exercise during infection seems to create an unfavourable condition for proper tissue healing. In our study, the immunohistochemical characterisation of the inflammatory lesions confirms this assumption. In exercising mice, higher cellularity of the remaining inflammatory lesions was observed. Detailed analysis showed a particular increased presence of CD8-positive T cells, a cell type responsible for viral clearing early in the infection, but on the other hand, this cell type is associated with an increased risk of autoimmune reactivity and a detrimental further disease course^39^. Additionally, the number of pro-inflammatory iNOS-reactive macrophages was higher in exercising mice. This cell type is associated with more severe cardiac damage and subsequent remodelling^40^.

While blinded histological evaluation showed a difference in presence of reactive fibrosis patterns between exercising and sedentary mice, quantification of myocardial collagen content using image analysis software could not detect such differences. Automated software detection has its limitations, since it cannot differentiate between ‘physiological’ (*e.g.* vessel adventitia) and ‘pathological’ collagen. It can also not differentiate between patterns of fibrosis (fine interstitial strands versus scars with thick collagen bundles), while these different patterns are of pathophysiological importance. Moreover, the quantification of myocardial collagen content was performed on just one cross-section, and represents only a marginal fraction of the total cross-sectional area, even if prominent scars are present.

The absence of upregulation of collagen- and fibrosis-related genes in the CVB groups, despite manifest myocardial fibrosis on histology, is presumably related to the timing of measurement (42 days after inoculation). This is supported by others, who reported rapid return to baseline of collagen gene expression levels in murine viral myocarditis^41,42^. Concerning the genes related to fibrogenesis, in-house data have shown that CTGF gene expression is increased approximately sevenfold in male CVB mice 7 days post inoculation, but quickly normalises thereafter. This corresponds to previous literature, and similar findings have been reported for TGF-β^41,43^. Also MMPs are activated within hours to days after cardiac damage, with expression returning to baseline within the first weeks^41,44^. Finally, in viral myocarditis in BALB/c mice (which progress to chronic myocarditis), expression of genes associated with cardiovascular remodelling during the acute phase is largely absent in the chronic stage of disease^45^. Altogether, these elements indicate that the majority of fibrotic remodelling occurs relatively early in the course of disease, necessitating further exploration of these time-dependent changes^46,47^.

Both the reactive type of fibrosis as well as the replacement type of fibrosis are important prognostic determinants. MF reduces chamber compliance, comprises tissue oxygenation and promotes reentrant tachycardias via slowing or blocking of conduction^48^. We are the first to assess arrhythmogenicity in a viral myocarditis model in vivo. Although the impact of exercise on arrhythmia inducibility and burden were not significant, the highest inducibility and cumulative burden were observed in the CVB-EEX group. The number of observations and complexity of ventricular stimulation protocols in mice preclude a definitive answer, but the findings are in line with the known impact of ventricular fibrosis on arrhythmogenesis. Further study (also modulating sports-related factors like duration, intensity and timing) will be needed to elucidate the proarrhythmic relevance of the increased fibrosis seen in our exercising infected mice. It is known that exercise is proarrhythmogenic in the context of other cardiac disease (e.g. arrhythmogenic cardiomyopathy)^49^. Interestingly, our noninfected control exercise group experienced a relatively high arrhythmia burden. Benito et al. have described promotion of ventricular tachycardia inducibility after 16 weeks of vigorous running training in healthy rats, with concomitant collagen deposition in the right ventricle as potential substrate^50^. Myocarditis and exercise combined could potentially lead to additive arrhythmia burden, which is of manifest clinical importance in a world in which many engage vigorously in endurance exercise while not always paying attention to viral symptoms.

The mechanisms by which exercise affects the course of viral myocarditis and its associated fibrogenesis are currently unclear. One potential mechanism is the higher pressure during exercise and concomitant wall stresses^1,51^. Pressure overload is a known stimulus for reactive fibrosis^46^. However, as exercise imposes a disproportionate load on the RV compared to the LV, one would expect the RV to be disproportionately affected by pathological fibrotic remodelling^9^. In contrast, we observed a uniform increase of fibrosis in the heart sections, and a scar distribution with rather a tendency to the left ventricle. Another mechanism that is often put forward is that of immune system modulation by exercise. The interaction between exercise and immunity is intricate, and the effects of exercise on immune function are probably dependent on different exercise variables (such as modality, duration, intensity and training scheme) and the time interval after the exercise bouts^11,14,15,18^. Our results confirm a different immune response (increased presence of CD8^+^ T lymphocytes and pro-inflammatory macrophages) in exercising animals, a mechanism which certainly deserves further exploration.

Our findings in exercising mice might be a homologue of what happens in athletes, i.e., that their training activities make them more prone for fibrosis and arrhythmias in the context of a subclinical myocarditis but that discontinuation of training during infection might be beneficial, as is recommended in the current guidelines^12^. It is however important to recognise that we used an animal model to study the effect of a certain combination of pre-inoculation and post-inoculation training duration, intensity and type of exercise. Apart from different aspects of exercise itself, the strains of mice and virus, as well as the dose and route of inoculation may all impact the possible interaction between exercise, myocarditis, fibrosis and arrhythmogenicity, and require further study.

## Study limitations

This study was limited to male mice. A sex difference has been observed in viral myocarditis, both in men and mice, in which male sex is more prone to myocarditis and MF, and confers worse prognosis^6,52–54^. Therefore we chose to study male mice as this is more clinically relevant (most athletes with arrhythmias are men) and would potentially give more clear-cut results. In addition, we wanted to avoid an impact of the menstrual cycle on the different aspects of the study (*i.a.* exercise capacity, immunity, and myocarditis).

Semiquantitative histopathological scoring represents a widely used and accepted method in preclinical and clinical studies, including myocarditis research^30,55^. It offers the advantage that the expert evaluator adds insight into the pathobiological aspects of the disease, rather than relying on undifferential computer quantification. We have prevented bias by paying rigorous attention to blinding and using a consensus score from 2 independent evaluators.

Inherent to the disease model and study design, two forms of ‘survivorship bias’ may have occurred. Firstly, the most severe myocarditis cases with the highest fibrogenic potential could have been excluded from analysis because of premature death. However, deceased animals showed similar degrees of myocardial inflammation and fibrosis compared to animals sacrificed at the same stage of disease (in the context of another study). For practical reasons, our study was run in two cohorts, following the same study protocol. The fact that in one of both cohorts, the mortality in the CVB-EEX and CVB-SED groups was identical (1/11 animals), and results followed the same trend as for both cohorts combined, additionally argues against substantial survivorship bias due to mortality. The second form of survivorship bias relates to the exclusion of exercising CVB animals if they could not adhere to the training protocol. At this point, it is not possible to identify the causes of exercise incompliance in mice (psychological reasons have been described, and the possibility of cardiocirculatory limitations seems evident in myocarditis). Again, histological analysis did not demonstrate notable differences in inflammation or fibrosis in these animals compared to their cagemates, and the number of exclusions were low.

As mentioned in the Discussion section, forced treadmill running is often the preferred training modality in preclinical research because of its ability to provide precise control over exercise intensity and duration. However, several factors related to forced running training, such as handling procedures, exposure to the apparatus, noxious running stimuli, and disruption of the day/night cycle, could be stressful for rodents. Although we attempted to minimise and equalise stress across the groups, and excluded animals who received excessive footshocks, we cannot exclude the possibility that the exercise groups were exposed to higher stress levels compared to sedentary animals.

## Conclusion and future perspectives

Our animal experiments confirm clinical observations that continued exercise during viral myocarditis, but not the athletic status per se (cf. pretraining), might establish an unfavourable environment for adequate cardiac healing, and facilitate the formation of myocardial fibrosis in this context. As outlined above, several questions remain unanswered concerning such viral myocarditis/exercise interaction, with multiple variables of exercise, virus and mouse strain to be explored. Our immunohistochemical characterisation of the inflammatory infiltrate suggest a role for exercise-associated immune susceptibility^14^. Murine models have emphasised the importance of genotype for the outcome of viral myocarditis^38^. Although the mice of our study might be more genetically homogeneous then athletes, it suggests the likelihood of even more complex interactions between genes, infection and exercise that define susceptibility to arrhythmias.

## Clinical perspectives

### Competency in medical knowledge

Diagnostic work-up of athletes frequently reveals nonischaemic myocardial fibrosis. The fibrosis is often considered as sequelae of subclinical myocarditis, and is known to be associated with ventricular arrhythmias. This study is the first to provide evidence that endurance exercise modulates the inflammatory reaction to viral myocarditis, facilitates fibrotic transformation (albeit without persistent fibrogenetic gene upregulation), and as such, may promote ventricular arrhythmias. This work adds to our knowledge on the intricate relationship between viral myocarditis and exercise, and contributes to the unravelling of the clinical conundrum of isolated myocardial fibrosis in athletes.

### Translational outlook

Here we investigate for the first time the impact of endurance exercise on myocardial fibrosis and ventricular arrhythmogenicity in the context of viral myocarditis. The data support the hypothesis that athletes may be predisposed to worse outcomes in viral myocarditis. Further study of the variables involved, like type, intensity and timing of sport is required.

### Supplementary Information

Supplementary Information 1.

Supplementary Information 2.

## Data Availability

The data underlying this article (including histology images and electrophysiology tracings) will be shared on reasonable request to the corresponding author.
