# Supplementary material for: The influence of endurance exercise training on myocardial fibrosis and arrhythmogenesis in a coxsackievirus B3 myocarditis mouse model
Source: Sci Rep. 2024 Jun 2;14:12653. doi: 10.1038/s41598-024-61874-x (PMC11144711; doi:10.1038/s41598-024-61874-x)
Supplement: Supplementary file 2 — Supplementary Information 2. [file 41598_2024_61874_MOESM2_ESM.docx]

# **The influence of endurance exercise training on myocardial fibrosis and arrhythmogenesis in a coxsackievirus B3 myocarditis mouse model**

Kasper Favere^1,2,3,4*^, Manon Van Hecke^5*^, Sander Eens^1,2^, Matthias Bosman^1^, Peter L. Delputte^6^, Johan De Sutter^4^, Erik Fransen^7^, Tania Roskams^5†^, Pieter-Jan Guns^1†^, Hein Heidbuchel^2,3†^

^1^ Laboratory of Physiopharmacology, GENCOR, University of Antwerp, 2610 Antwerp, Belgium
^2^ Research Group Cardiovascular Diseases, GENCOR, University of Antwerp, 2610 Antwerp, Belgium
^3^ Department of Cardiology, Antwerp University Hospital, 2650 Antwerp, Belgium
^4^ Department of Internal Medicine, Ghent University, 9000 Ghent, Belgium
^5^ Translational Cell & Tissue Research, Department of Imaging & Pathology, University of Leuven, 3000 Leuven, Belgium
^6^ Laboratory of Microbiology, Parasitology and Hygiene, University of Antwerp, 2610 Antwerp, Belgium
^7^ Centre for Medical Genetics, University of Antwerp, 2610 Antwerp, Belgium

*Shared first authorship
†Shared senior authorship

## Running title

Myocarditis in running mice

## Correspondence

Kasper Favere
Department of Cardiology
Antwerp University Hospital
Drie Eikenstraat 655
2650 Antwerp
Belgium

Email: Kasper.Favere@uantwerpen.be, Kasper.Favere@ugent.be. Tel. +32 3 821 35 38.
ORCID iD: https://orcid.org/0000-0003-1848-3701

# **Supplementary Figures**

## Supplementary Figure 1 | Study design

(A) Continued EEX study. Mice are allocated to four groups. Exercise (EEX) groups receive exercise training throughout the entire study protocol (green bar). The coxsackievirus (CVB) groups are inoculated with coxsackievirus B3 (CVB) at day 0 (D0) and develop acute, self-limiting viral myocarditis (VMC)(orange triangle). Exhaustion testing (EXH) is performed at the start of the study, prior to inoculation and at the end of the study. At sacrifice (SACR), electrophysiology studies (EPS) were performed in a selection of the animals. Hearts were collected from all animals alive upon completion of the study. (B) Pretrained EEX study. Mice were allocated to one of 4 groups. Pretrained groups (preEEX) were subjected to a 13-week exercise programme, whereas sedentary groups (preSED) were not. After 13 weeks with (preEEX) or without (preSED) exercise training, mice were injected with either CVB to induce VMC, or with vehicle (PBS). Electrophysiology studies were performed immediately prior to sacrifice.

## Supplementary Figure 2 | Myocarditis severity scoring system

Based on the location of the inflammatory infiltrate and the presence and extent of myocyte loss, a myocarditis severity score was assigned in a blinded fashion for each mouse heart. The following scores were considered: absence of inflammation, perivascular inflammatory infiltration, interstitial inflammatory infiltration, interstitial inflammatory infiltration with focal cell loss, interstitial inflammatory infiltration with confluent cell loss. Additionally, the location of each lesion was specified (left or right ventricle, subendocardial, midmyocardial of subepicardial).

## Supplementary Figure 3 | Myocardial fibrosis scoring system

According to the aetiology of fibrosis, different categories were considered during blinded scoring of each mouse heart: absence of fibrosis, perivascular (PV) fibrosis by which is meant the area surrounding the larger blood vessels (limited (<50% of vessels involved) or extensive (>50% of vessels involved)), interstitial (IS) fibrosis by which is meant the space in between individual cardiomyocytes (limited or extensive) and myocardial scarring by which is meant a continuous patch of fibrosis without intermittent cardiomyocytes (absent or present, in which case an absolute number was counted). Additionally, the location of each lesion was specified (left or right ventricle, subendocardial, midmyocardial of subepicardial).

## Supplementary Figure 4 | Temporal evolution of myocardial inflammation

The images on the left-hand side illustrate the appearance of inflammatory lesions observed 7 days after inoculation in this murine model, and are shown for reference. At this early stage of disease, inflammatory lesions are highly cellular with abundant presence of macrophages. Multiple sites show cardiomyocytes with necrotic appearance. The images on the right-hand side represent typical lesions encountered in CVB-SED animals sacrificed at D43-50 after inoculation in the context of this study. These are characterised by reduced presence of immune cells, and appearance of haemosiderin-laden macrophages and spindled fibroblasts.

## Supplementary Figure 5 | Myocardial scar location in the continued EEX study

Distribution of myocardial scars within the CVB-EEX group (top) and CVB-SED group (bottom). Within the left ventricle (LV), 4 regions were identified: anterior wall (ant.), lateral wall (lat.), inferior wall (inf.) and septum. The right ventricular (RV) free wall was considered a fifth region. In each of these 5 regions, scars could be located in the subendocardial, midmyocardial or subepicardial zone. The intensity of the red colour correlates with the scar prevalence in each of the areas.

## Supplementary Figure 6 | Arrhythmia duration in the continued EEX study

Beat count of individual ventricular arrhythmia episodes. For all animals completing the entire stimulation protocol, the duration of each of the (NS)VT episodes elicited is shown as a data point for that animal (which have been given arbitrary identifier numbers). Animals in which no arrhythmias could be elicited, received the value zero.

## Supplementary Figure 7 | Graphical abstract

This study investigated the effect of endurance exercise on myocardial fibrosis development in viral myocarditis. Mice with experimentally induced viral myocarditis were randomised to treadmill training (from 2 weeks before virus inoculation till sacrifice at 6 weeks) or not. Exercise was associated with a more pro-inflammatory cell infiltrate, and increased interstitial fibrosis (P=0.049). The cumulative duration of induced ventricular arrhythmias paralleled the increased fibrosis but the difference did not reach significance (P=0.084).

## **Supplementary Figure 1: Study design**

**
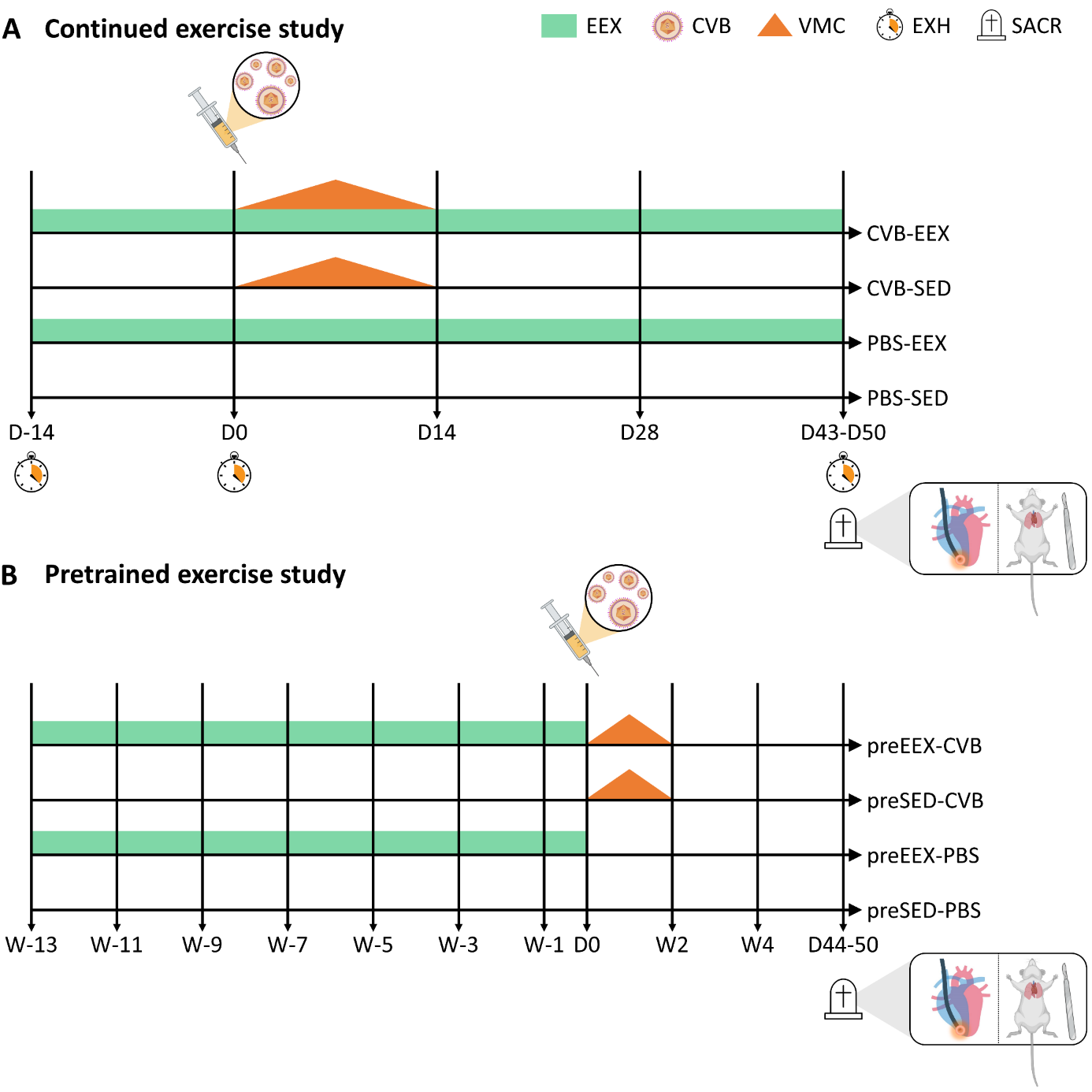
**

## **Supplementary Figure 2: Myocarditis severity scoring system**


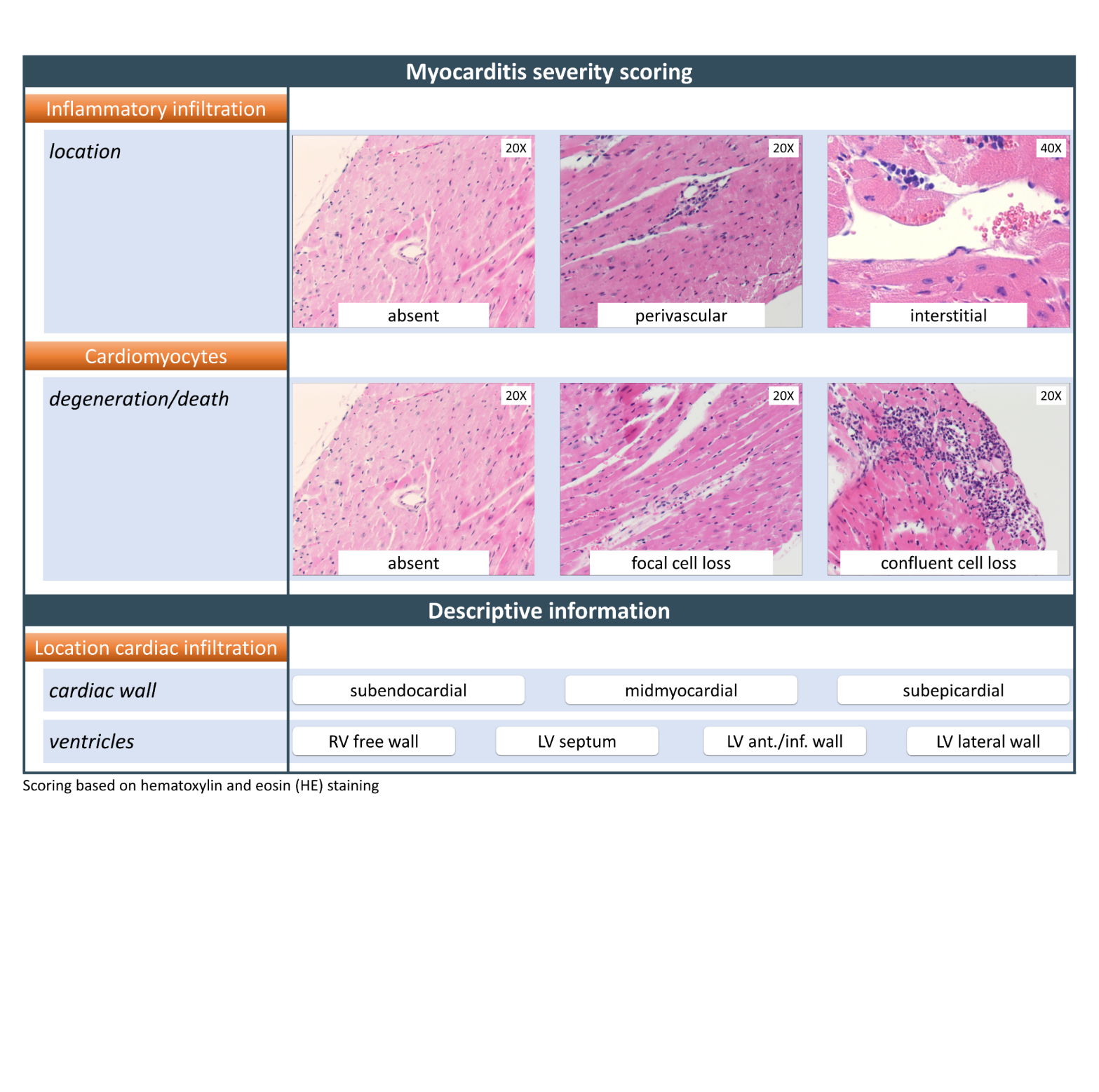


## **Supplementary Figure 3: Myocardial fibrosis scoring system**


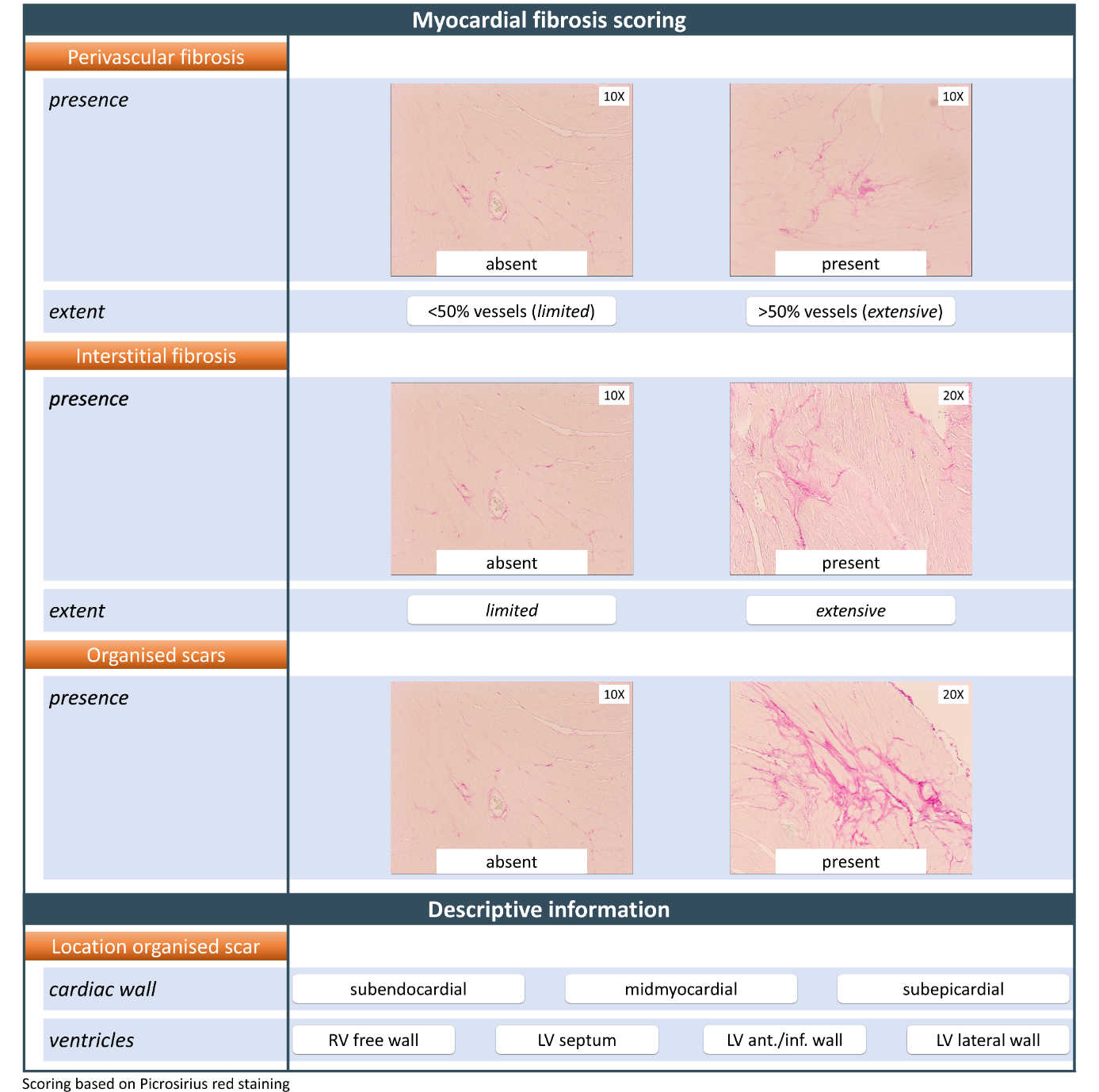


## **Supplementary Figure 4: Temporal evolution of myocardial inflammation**


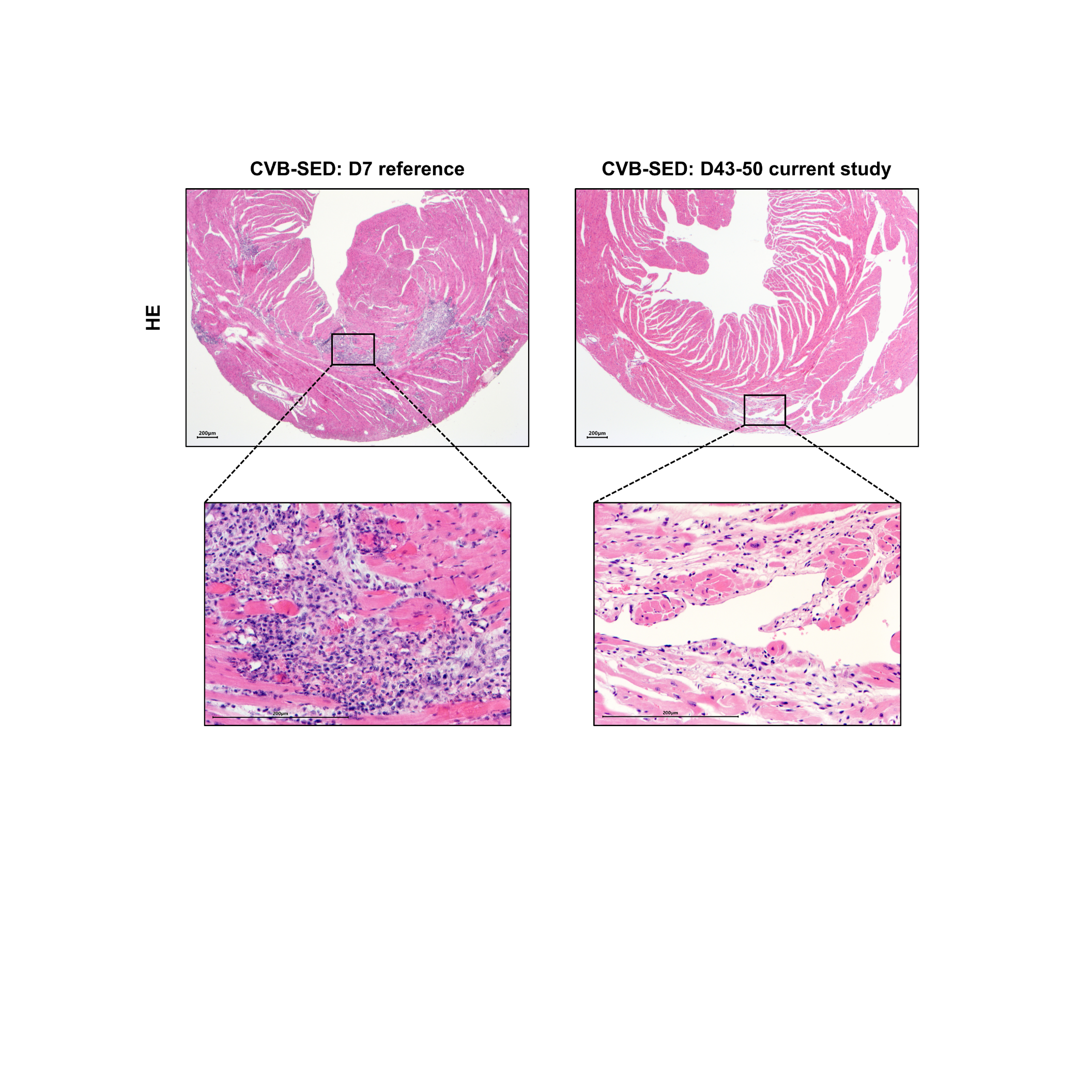


## **Supplementary Figure 5: Myocardial scar location in the continued EEX study**


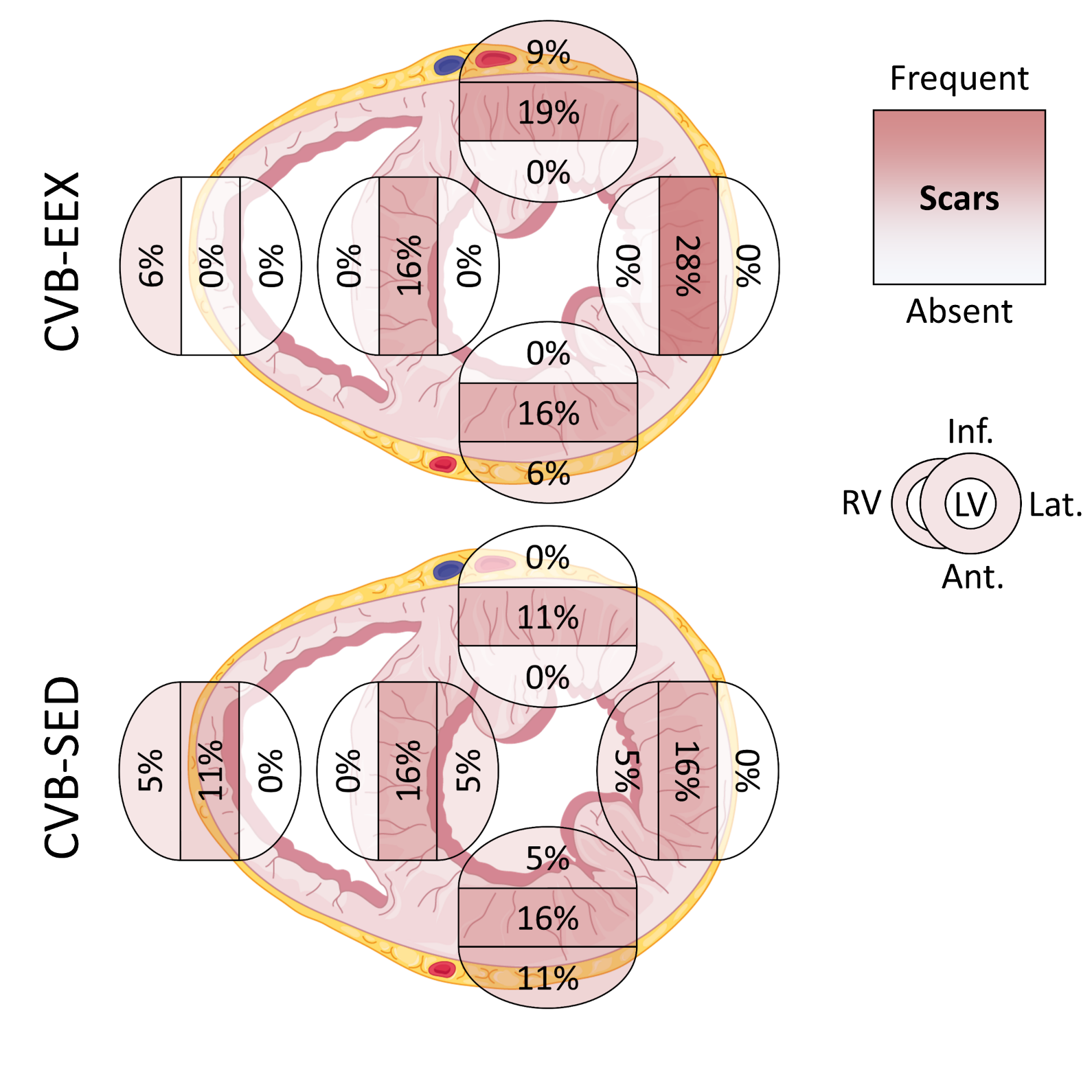


## **Supplementary Figure 6: Arrhythmia duration in the continued EEX study**

**
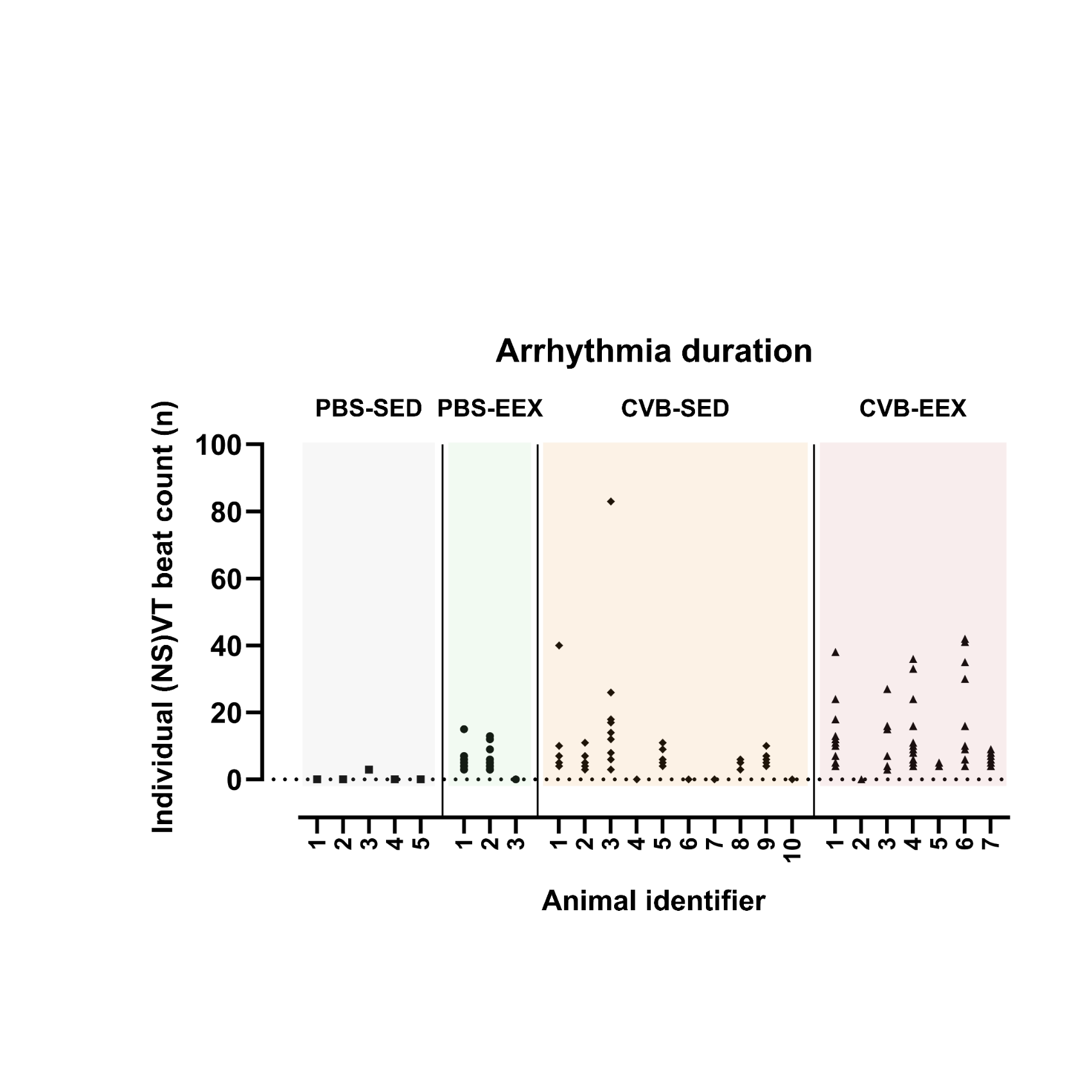
**

## **Supplementary Figure 7: Graphical abstract**


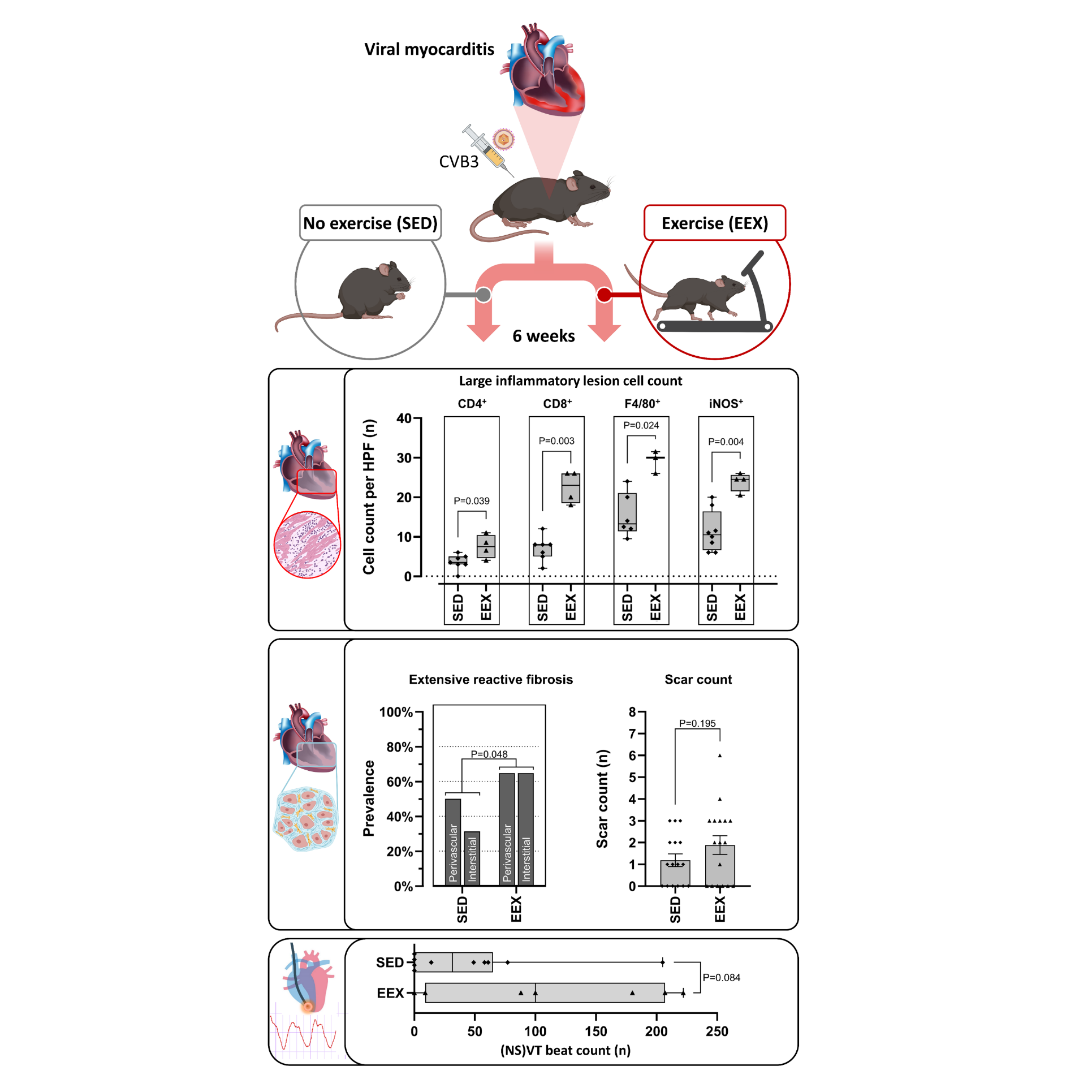


# **Supplementary Tables**

## Supplementary Table 1A | Survival analysis continued EEX study: number alive at each timepoint

| **Group** | **D0** | **D6** | **D8** | **D9** | **D10** | **D14** | **D15** | **D43-D50** |
| --- | --- | --- | --- | --- | --- | --- | --- | --- |
| PBS-SED | 14 |  |  |  |  |  |  | 14 |
| PBS-EEX | 14 |  |  |  |  |  |  | 14 |
| CVB-SED | 22 |  |  | 22 (90.9% [0.797-1]) | 20 (81.8% [0.672-0.996]) | 18 (77.3% [0.616-0.969]) | 17 (72.7% [0.563-0.939]) | 16 |
| CVB-EEX | 19 | 19 (94.7% [0.852-1]) | 18 (89.5% [0.767-1]) |  |  |  |  | 17 |
| Data correspond to Figure 1B. Data are expressed as number at risk (% survival [95% confidence interval]). | | | | | | | |  |

## Supplementary Table 1**B** | Survival analysis pretrained EEX study: number alive at each timepoint

| **Group** | **D0** | **D7** | **D10** | **D26** | **D44-D50** |
| --- | --- | --- | --- | --- | --- |
| preSED-PBS | 5 |  |  |  | 5 |
| preEEX-PBS | 4 |  |  |  | 4 |
| preSED-CVB | 10 | 10 (90% [0.714-1) | 9 (80% [0.553-1]) | 8 (70% [0.416-0.984]) | 7 |
| preEEX-CVB | 9 |  |  |  | 9 |
| Data correspond to Figure 1C. Data are expressed as number at risk (% survival [95% confidence interval]). | | | | |  |

## Supplementary Table 2A | Arrhythmia inducibility in the continued EEX study: number at risk at each stimulation step

| **Group** | **Start** | **Isoprenaline** | | | |
| --- | --- | --- | --- | --- | --- |
|  |  | **Burst 1** | **Ramp 1** | **Ramp 2** | **Burst 2** |
| PBS-SED | 5 |  |  |  | 5 (80.0% [0.516-1]) |
| PBS-EEX | 5 | 4 (50.0% [0.188-1]) |  | 2 | 1 |
| CVB-SED | 10 | 10 (60.0% [0.362-0.995]) | 6 (50.0% [0.269-0.929]) | 5 (40.0% [0.187-0.855]) | 4 |
| CVB-EEX | 9 | 9 (44.4% [0.214-0.923]) |  | 4 (33.3% [0.132-0.840]) | 2 (16.7% [0.032-0.882]) |
| Data correspond to Figure 7B. Data are expressed as number at risk (% survival [95% confidence interval]). | | | | | |

## Supplementary Table 2B | Arrhythmia inducibility in the pretrained EEX study: number at risk at each stimulation step

| **Group** | **Start** | **Isoprenaline** | | | |
| --- | --- | --- | --- | --- | --- |
|  |  | **Burst 1** | **Ramp 1** | **Ramp 2** | **Burst 2** |
| preSED-PBS | 5 | 5 (80.0% [0.449-1]) | 3 (53.3% [0.044-1]) |  | 2 |
| preEEX-PBS | 4 | 4 (75% [0.325-1]) |  |  | 3 |
| preSED-CVB | 6 | 6 (50% [0.100-0.900]) |  |  | 3 |
| preEEX-CVB | 7 | 7 (85.7% [0.598-1]) | 6 | 5 (68.6% [0.321-1]) | 4 |
| Data correspond to Figure 7D. Data are expressed as number at risk (% survival [95% confidence interval]). | | | | | |

## Supplementary Table 3 | Studies evaluating the effect of exercise on viral myocarditis

| **First Author, Year (Ref)** | **Virus – Route – Dose** | **Mice (Age)** | **Exercise** | | **Viral load** | | **Histopathology** | | **Cardiac function** | **Mortality** |
| --- | --- | --- | --- | --- | --- | --- | --- | --- | --- | --- |
|  |  |  | **Intervention (EEX)** | **Control** | **Myocardium** | **Serum** | **Inflammation** | **Fibrosis** |  |  |
| Federici, 1963^[1]^ | CVA9 (strain 13) – IP – 10^5^ TCID50 | ♂ C3H (8-9 months) | Spontaneous running in activity wheels:  - prior inoculation: three 2h-periods at intervals 2-3 d; followed by 4 50-min periods at intervals 1-2 days - after inoculation: 50 min daily until D6 PI; subsequently 2-4x/week until D106 PI | No activity wheels | D6 & 9 PI: 7/13 (54%)(CVA-EEX) vs. 8/12 (67%)(CVA) virus positive |  | D6 & 9 PI: 5/13 (38%)(CVA-EEX) vs. 3/12 (33%)(CVA) myocarditis |  |  | No comparison |
| Tilles, 1964^[2]^ | CVA9 (strain 13) – IP – 10^6^ TCID50 | ♂ C3H (10 months) | Swimming:  - prior inoculation: 1x/d for 5 min (D7), 15 min (D6), 30 min (D5), 60 min (D4); 2x/d for 60 min D3-D1 - after inoculation: 2x/d 45 min (first 3 days); 2x/d 40 min (until sacrifice) | Not exercised | D1 PI: 2/11 (18%)(200 & <100 PFU/mL)(CVA-EEX) vs. 4/11 (36%)(1700, 600, 200, <200 PFU/mL)(CVA) D4 PI: 6/11 (55%)(120000, 61000, 60000, 30000, 24000, 2600 PFU/mL)(CVA-EEX) vs. 1/12 (8%)(7000 PFU/mL)(CVA) D9 PI: 4/10 (40%)(15000, 4500, 400, <100 PFU/mL)(CVA-EEX) vs. 1/12 (8%)(<100 PFU/mL)(CVA) | D1 PI: 7/11 (64%)(CVA-EEX) vs. 8/11 (73%)(CVA) D4 PI: 1/12 (8%)(CVA-EEX) vs. 2/12 (17%)(CVA) D9 PI: 0/11 (0%)(CVA-EEX) vs. 0/12 (0%)(CVA) | D4 PI: 1/11 (9%)(CVA-EEX) vs. 1/12 (8%)(CVA) D9 PI: 2/10 (20%)(CVA-EEX) vs. 0/12 (0%)(CVA) |  |  | No comparison |

| Gatmaitan, 1970^[3]^ | CVB3 (Nancy strain) – IP & IC - 7.5 x 10³ PFU (IP) & 3 x 10³ (IC) | Swiss ICR (14 days) | Swimming until drowning (1x/d and from 19^th^ day of life 2x/d): - immediate (IMM): continually - delayed (DEL): from D9 PI until sacrifice | Not exercised | D3 PI: 6/6 (100%)(IMM) vs. 6/6 (100%)(DEL) vs. 6/6 (100%)(control); equal titer (± 10^4^ PFU/g)  D6 PI: 6/6 (100%)(IMM) vs. 4/6 (67%)(DEL) vs. 4/6 (67%)(control); IMM swimming titer x 530 compared to DEL and control D9 PI: 3/6 (50%)(IMM) vs. 2/6 (33%)(DEL) vs. 0/6 (0%)(control); IMM swimming titer x 100 compared to DEL and control D13 PI: 0/6 (0%)(IMM) vs. 0/6 (0%)(DEL) vs. 0/6 (0%)(control)  D20 PI: 0/3 (0%)(IMM) vs. 0/6 (0%)(DEL) vs. 0/6 (0%)(control)  D40 PI: 0/3 (0%)(IMM) vs. 0/6 (0%)(DEL) vs. 0/6 (0%)(control) | D3 PI: 6/6 (100%)(IMM) vs. 6/6 (100%)(DEL) vs. 5/6 (83%)(control); no difference in titer  D6 PI: 4/6 (67%)(IMM) vs. 2/6 (33%)(DEL) vs. 2/6 (33%)(control); no difference in titer D9 PI: 0/6 (0%)(IMM) vs. 0/6 (0%)(DEL) vs. 0/6 (0%)(control)  D13 PI: 0/6 (0%)(IMM) vs. 0/6 (0%)(DEL) vs. 0/6 (0%)(control)  D20 PI: 0/6 (0%)(IMM) vs. 0/6 (0%)(DEL) vs. 0/6 (0%)(control)  D40 PI: 0/6 (0%)(IMM) vs. 0/6 (0%)(DEL) vs. 0/6 (0%)(control) | Severe myocarditis from D13 PI: 8/12 (67%)(IMM) vs. 5/18 (28%)(DEL) vs. 0/17 (0%)(control) |  |  | 15/30 (50%)(IMM) vs. 5/36 (13.9%)(DEL) vs. 2/36 (5.5%)(control) |
| --- | --- | --- | --- | --- | --- | --- | --- | --- | --- | --- |
| Reyes, 1981^[4]^* | CVB3 | (14 days) | Swimming: 30 min/d during D0-9 PI | No exercise intervention |  |  |  |  |  | 45% vs. significantly lower |
| Kiel, 1989^[5]^ | CVB3 – IP –1.0 x 10²^.1^ TCID50 | ♂ C3H (4 weeks) | Swimming: 60 min/d during D1-D9 PI | No exercise intervention | D1 PI: absent D3 PI: 3.85 log_10_/0.1 mL vs. 3.99 log_10_/0.1 mL D6 PI: 5.71 log_10_/0.1 mL vs. 4.95 log_10_/0.1 mL (significant) D9 PI: 5.37 log_10_/0.1 mL vs. 4.33 log_10_/0.1 mL (significant) D14 PI: 1.96 log_10_/0.1 mL vs. 1.37 log_10_/0.1 mL | 24h PI: no difference in titer 72h PI: higher titer exercise vs. control | Massive calcium deposition and more dilation exercise vs control D1 PI: no myofiber destruction D3 PI: no myofiber destruction D6 PI: 26-50% involvement exercise, no difference vs. control D9 PI: >75% involvement exercise, no difference vs. control D13 PI: myocardial histopathologic grade 3.6 exercise vs. 2.0 control |  |  | 23% vs. 5.9% |
| Ilbäck, 1989^[6]^ | CVB3 (Nancy strain) – IP – 2 x 10^4^ infectious particles | ♀ Balb/c CUM (8-14 weeks) | Treadmill running (70 minutes – majority 30 cm/s):  - immediate (IMM): 0h PI - delayed (DEL): 48h PI | No exercise intervention |  |  | Inflamed area: 4.77% (IMM) vs. 7.85% (DEL) vs. 4.32% (control) |  |  | No difference |
| Cabinian, 1990^[7]^* | CVB3 – IP – 10(2.5) TCID50 | ♂ C3H/HeN (3 weeks) | Swimming: D1-9 | No exercise intervention |  |  |  |  |  | 18/25 (72%) vs. 1/25 (4%) |
| Abbreviations: CVA, coxsackievirus group A; CVB, coxsackievirus group B; EEX, exercise; IC, intracerebrally; IP, intraperitoneal; PI, post inoculation; TCID50, median tissue culture infectious dose. *Full text could not be obtained. | | | | | | | | | | |

# **Supplementary Methods**

## Virus culture

Human coxsackievirus B3 (Nancy strain) was purchased from American Tissue Culture Collection (ATCC, France). Vero cells (ATCC, France) were used as propagation host and grown to 80-90% confluency in a culture flask. The cells were cultured in minimum essential media (MEM) (Gibco, Waltham MA, USA) containing 10% inactivated fetal bovine serum (FBS)(Gibco, Waltham MA, USA) and 2% penicillin/streptomycin (Gibco, Waltham MA, USA). Prior to inoculation, the growth medium was removed and the cells washed with sterile PBS (Gibco, Waltham MA, USA). The virus was dissolved in a small volume of serum-free MEM to have sufficient inoculum for uniform infection throughout the cell monolayer. To allow viral adsorption, the flask was placed in the incubator (37°C, 5% CO_2_) for 1 hour. Subsequently, the adsorption process was ended by adding MEM with 2% FBS. After 48 h of incubation (37°C, 5% CO_2_), the cell culture medium (now containing the grown virus) was collected and centrifuged for 10 min at 5250 RPM and at 4°C. The supernatant (concentrated virus) was aliquoted and stored at -80°C until further use.

## Plaque assay

Vero cells were plated in a 6-well plate in monolayer with 90-100% confluency. A 10-fold serial dilution series of the virus was prepared using MEM with 2% penicillin/streptomycin as diluent. After washing with serum-free MEM, the wells were inoculated with a chosen dilution of the dilution series. MEM containing 2% penicillin/streptomycin was used as negative control, and a very low dilution of the virus stock as positive control. The virus solution was left to incubate for 1 hour during which the plate was gently rocked every 15 minutes to uniformly distribute the inoculum over the monolayer. Subsequently, the virus medium was removed and the wells were overlayed with warmed 0.6% Avicel (FMC BioPolymer, Philadelphia, PA, USA) in MEM with 10% FBS to control viral spread within the monolayer.^[8,9]^ Movement of the plate during incubation was minimised. After 2 days, the Avicel overlay was removed and 4% paraformaldehyde (Merck, Darmstadt, Germany) solution was applied for 20 minutes to fixate the cells. Afterwards, the fixative was discarded and the wells were thoroughly rinsed with water to remove any residual overlay or fixative solution. The wells were stained by covering them with 0.25% crystal violet (Merck, Darmstadt, Germany) solution for 15 minutes. Virus titres are expressed as the mean plaque forming units (PFU) per mL of virus solution.

# **References**

1 Federici, E. E., Lerner, A. M. & Abelmann, W. H. Observations on the course of Coxsackie A-9 myocarditis in C3H mice. *Proc. Soc. Exp. Biol. Med.* **112**, 672-676 (1963). <https://doi.org/10.3181/00379727-112-28136>

2 Tilles, J. G. *et al.* Effects of Exercise on Coxsackie A9 Myocarditis in Adult Mice. *Proc. Soc. Exp. Biol. Med.* **117**, 777-782 (1964). <https://doi.org/10.3181/00379727-117-29696>

3 Gatmaitan, B. G., Chason, J. L. & Lerner, A. M. Augmentation of the virulence of murine coxsackie-virus B-3 myocardiopathy by exercise. *J. Exp. Med.* **131**, 1121-1136 (1970). <https://doi.org/10.1084/jem.131.6.1121>

4 Reyes, M. P., Ho, K. L., Smith, F. & Lerner, A. M. A Mouse Model of Dilated-Type Cardiomyopathy Due to Coxsackievirus-B3. *Journal of Infectious Diseases* **144**, 232-236 (1981). <https://doi.org/DOI> 10.1093/infdis/144.3.232

5 Kiel, R. J., Smith, F. E., Chason, J., Khatib, R. & Reyes, M. P. Coxsackievirus B3 myocarditis in C3H/HeJ mice: description of an inbred model and the effect of exercise on virulence. *Eur. J. Epidemiol.* **5**, 348-350 (1989). <https://doi.org/10.1007/BF00144836>

6 Ilback, N. G., Fohlman, J. & Friman, G. Exercise in coxsackie B3 myocarditis: effects on heart lymphocyte subpopulations and the inflammatory reaction. *Am. Heart J.* **117**, 1298-1302 (1989). <https://doi.org/10.1016/0002-8703(89)90409-2>

7 Cabinian, A. E. *et al.* Modification of Exercise-Aggravated Coxsackievirus-B3 Murine Myocarditis by Lymphocyte-T Suppression in an Inbred Model. *Journal of Laboratory and Clinical Medicine* **115**, 454-462 (1990).

8 Matrosovich, M., Matrosovich, T., Garten, W. & Klenk, H. D. New low-viscosity overlay medium for viral plaque assays. *Virology journal* **3**, 63 (2006). <https://doi.org/10.1186/1743-422X-3-63>

9 Mendoza, E. J., Manguiat, K., Wood, H. & Drebot, M. Two Detailed Plaque Assay Protocols for the Quantification of Infectious SARS-CoV-2. *Curr. Protoc. Microbiol.* **57**, ecpmc105 (2020). <https://doi.org/10.1002/cpmc.105>
